# Facilitation in the Dry Season: Species Interactions Between a Limestone-Endemic Plant and Moss Altered by Precipitation Dynamics

**DOI:** 10.3390/plants14162588

**Published:** 2025-08-20

**Authors:** Ali Raza, Shao-Jun Ling, Ya-Li Wei, Saraj Bahadur, Ming-Xun Ren

**Affiliations:** 1 Ministry of Education Key Laboratory for Genetics and Germplasm Innovation of Tropical Special Forest Trees and Ornamental Plants, Hainan University, Haikou 570228, China; aliraza5789@gmail.com (A.R.); sirajbahadur14@gmail.com (S.B.); 2 International Joint Center for Terrestrial Biodiversity Around South China Sea of Hainan Province, Hainan University, Haikou 570228, China

**Keywords:** *Oreocharis hainanensis*, moss *Leucobryum aduncum*, facilitation, competition, microbial community, physicochemical parameters

## Abstract

Plant-to-plant interactions are essential for structuring plant communities and supporting adaptation in nutrient-poor, seasonally dry environments. This study examined the interactions between moss *Leucobryum aduncum* Dozy & Molk and *Oreocharis hainanensis* by analyzing microbial communities and physicochemical parameters across various sample types. These included soil [bare (B), *O. hainanensis* (O), moss (M), and moss + *O. hainanensis* (MO)], rhizosphere soil [*O. hainanensis* (ORS), moss (MRS), and moss + *O. hainanensis* (MORS)], and root [*O. hainanensis* (OHR), moss (MR), and moss + *O. hainanensis* (MOR)] using metagenomics sequencing across dry and wet seasons in limestone habitats on Hainan Island. During the dry season, combined plant samples MOR, MO, and MORS showed higher nutrients, supported by microbes that enhance nutrient turnover, which may indicate facilitation. Conversely, during the wet season, increased moisture leads to decreased nutrient levels and microbial communities shift, associated with slower nutrient turnover in combined plant samples, which may reflect competition. According to KEGG analysis, an increase in oxidative phosphorylation and ABC transporters in the dry season supported the facilitative interaction, while quorum sensing and two-component systems supported the competitive interaction in the wet season. These findings show how shifts between facilitation and competition arise from seasonal conditions and microbes in the limestone ecosystem.

## 1. Introduction

Plant-to-plant interactions can be influenced by changing climate conditions. The modifications caused by these changes determine how plants respond to their surroundings. Some species may benefit from shifting conditions, while others struggle to survive. Environmental conditions influence how plants interact with microbes. Soil moisture, in conjunction with temperature and nutrient availability under diverse climatic conditions, can influence the composition and functional roles of the microbial community, thereby altering plant–plant relationships [1]. In particular, drought and moisture availability impact the microbial community in the soil, the root endosphere, and the rhizosphere [2]. Water availability increases microbial activity and nutrient cycling in the environment, whereas drought creates resource scarcity, which changes microbial composition and affects the nutrient dynamics [3]. The study of plant-to-plant interactions requires season-based examination for a complete understanding of these interactions. A variety of microorganisms can be found living in the soil, and these microbiomes are known to be critical indicators of plant health and have a significant impact on various plant health indicators that affect growth and production [4]. The crucial responsibilities of microorganisms include decomposing organic matter and fixing nitrogen while solubilizing phosphorus, which positively affects plant growth and their nutrient availability [5]. Nutrient-dependent alterations in plant-to-microbe interactions demonstrate the plant’s initiative in determining the plant-to-microbe association for optimized benefits. However, the interaction patterns between plants and microbes under extreme conditions like limestone environments are not thoroughly researched.

Limestone is characterized by scarce soil and a severe habitat, both of which have high levels of endemism. Still, there is currently a lack of comprehensive research on the colonization process and speciation mechanism of diversity on bare limestone. Additionally, Hainan Island has alternating dry and wet seasons, further highlighting the ecological dynamics [6]. In such environments, the species of plants play an essential role in determining the community structure, cycling of nutrients, and microbial dynamics. Understanding the interactions between the moss *L. aduncum* and a limestone-endemic plant, such as *O. hainanensis,* is essential for interpreting the plant coexistence mechanism and the microbial community’s role in response to these interactions on limestone.

Mosses, which act as pioneers in harsh habitats, have the potential to modify soil conditions because they affect water content and microbial ecosystem functions [7]. Mosses can influence the performance and dispersion of small or short-lived vascular plants through biotic and abiotic pathways, enhance the local habitat of exposed limestone, and establish colonization conditions for *Gesneriaceae* and other species [8]. Conversely, *O. hainanensis*, an endemic species on bare limestone, is a new species of *Gesneriaceae* that has adapted to the nutrient-deficient habitats and is described and illustrated from low-altitudinal karst areas on Hainan Island, South China. This new species is easily distinguished from its closely related *O. jasminina* by its campanulate floral tube, zygomorphic corolla, and exserted stamens [6]. The relationship between competitive and facilitative interactions between these species lacks understanding, especially concerning their impacts on microorganisms and nutrient cycling. The microbial communities in soils and rhizosphere soil function as vital regulators in plant relationships. Bacteria and fungi control important nutrient cycling processes and phosphorus solubilization with nitrogen fixation, affecting plant growth and resource availability [9]. However, there is still a knowledge gap regarding how microbial communities react to co-occurring plant species when different seasonal conditions prevail. This study on microbial effects in soil and rhizosphere soil and roots serves as a critical analysis because these microorganisms play a key role in plants through their control over nutrient cycles and plant responses [10]. Particularly, the root microbiome exerts immediate effects on nutrient absorption while affecting inter-species relations in co-occurring plant communities, and it strongly influences plant health [11]. Determining whether the plant-to-plant interaction is facilitative or competitive (positive or negative) is made easier by researching the microbial communities and nutrient dynamics in the soil, rhizosphere, and root.

**Figure 1:**
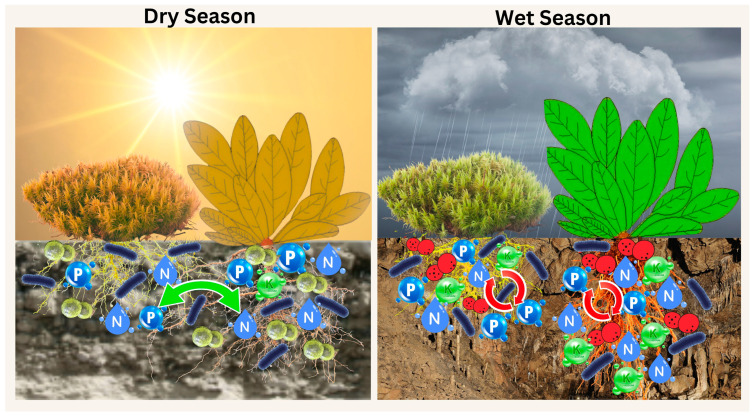
Illustration diagram of plant–plant interactions in the dry and wet seasons. The figure was created by the author using CANVA Pro software.

Competition can lead to the extinction of species, reduce biodiversity, and undermine the stability of ecosystems [12]. According to previous research, competition has a significant role in limiting a species’ geographic range, and it may also have an impact on how plants modify their distribution in response to potential future climate change [13]. However, facilitation can help to maintain diversity, especially in hostile situations where species usually depend on one another to survive [14]. A significant form of facilitative interactions is the phenomenon of nurse plants, where some plants (the “nurse”) facilitate the germination, development, and establishment of other plant species (the “beneficiary”) by providing shelter from abiotic stress and protection against biotic consumption, or both, which in turn might increase the richness of species in physically disturbed and/or stressful environments [15]. Considering the current scenario, this research examines the nature of the *O. hainanensis* and moss *L. aduncum* interaction, specifically whether it involves competition or facilitation, by analyzing the microbial community and nutrient cycling across soil, rhizosphere soil, and roots on limestone during both the dry and wet seasons. Thus, this research links the seasonal variation in microbes and nutrients to provide evidence for plant–plant interactions, offering insight into the availability of resources in nutrient-limited environments (Figure 1). We hypothesize that the co-occurrence of *O. hainanensis* and moss *L. aduncum* could alter microbial communities and physicochemical traits, reflecting shifts between facilitation and competition across seasons.

## 2. Results

### 2.1. Physicochemical Parameters

The physicochemical properties in the soil group (B-bare soil, O-*O. hainanensis* soil, M–moss *L. aduncum* soil, and combined plant sample MO–moss *L. aduncum* + *O. hainanensis* soil) during the dry and wet seasons changed dramatically. During the dry season, sample B possessed the lowest level of TN, while a single plant sample, O, demonstrated the highest TN. The level of TN in the single plant sample M was slightly lower than the single plant sample O and comparable to the combined plant sample MO. This suggests that the single plant sample O alone contributes more to the TN content as compared to the single plant sample M or the combined plant sample MO. The AN level was highest in the single plant samples O and B, while the single plant sample M had the lowest AN. The TP was relatively higher in the single plant sample M, followed by the combined plant sample MO. Conversely, the level of AP showed an increasing trend from sample B to sample MO, suggesting enhanced phosphorus availability when both plants coexist. Sample B showed the highest levels of TK and AK, while all plant-associated samples (O, M, and MO) contained less potassium. The combined plant sample MO had the highest OM and TC content, followed by the single plant sample M, and sample B had the lowest OM content.

**Figure 2:**
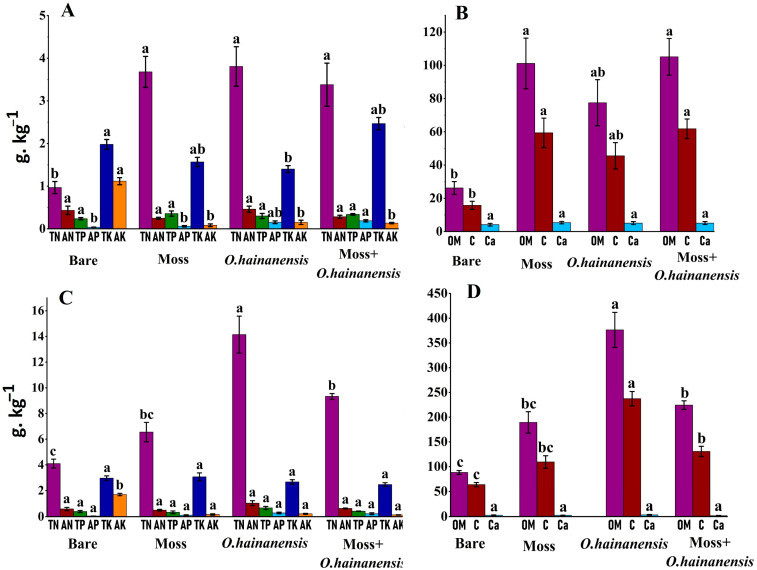
Soil physicochemical parameters among the four different groups in the dry and wet seasons. g·kg^−1^. AN: available nitrogen, TN: total nitrogen, OM: organic matter, C: total carbon, AP: available phosphorus, AK: available potassium, Ca^2+^: calcium ion, TP: total phosphorus, and TK: total potassium. The different superscript letters indicate significant differences at *p* < 0.05. All the nutrient values were converted from their measured units (mg/kg, %) to g·kg^−1^ for visual comparison. (**A**,**B**) Dry season (**C**,**D**) Wet season.

The wet season led to different patterns in nutrient dynamics than the dry season. Single plant sample O contained the greatest amount of TN, which was higher than the dry season, while the combined plant sample MO had lower TN levels. All samples demonstrated higher AN levels in comparison to the dry season, particularly in the single plant sample O, as well as in the combined plant sample MO. The TP content was higher in the single plant sample O than in the combined plant samples MO. Among all samples, the single plant sample O had the highest AP content, and the combined plant sample MO had a low AP content. The TK content and AK levels increased most significantly during the wet season, especially in the single plant sample M. All samples accumulated more OM and TC during the wet season, especially single plant sample O. All samples exhibited notably elevated SWC in the wet season compared to the dry season, particularly the single plant sample O. Overall, the research demonstrates that single plant species and their combinations, along with their seasonal growth cycles, each contribute to changing soil nutrient levels, with *O. hainanensis* generally showing stronger nutrient acquisition and transformation effects than moss *L. aduncum* (Table S1; Figure 2).

Similarly, the rhizosphere soil of a single plant sample, MRS–moss *L. aduncum* rhizosphere soil, *ORS-O*. *hainanensis rhizosphere soil*, and the combined plant sample MORS–moss *L. aduncum* + *O. hainanensis* rhizosphere soil during the dry season exhibited distinct variations in physicochemical properties. The TN was higher in the MRS, followed by the MORS. The AN content was highest in the ORS, while the MRS showed the lowest. This suggests that *O. hainanensis* might enhance nitrogen availability in the rhizosphere more effectively than moss *L. aduncum*. The TP was the highest in the MORS, while the MRS had the lowest TP content and the lowest AP.TK was the highest in the MRS (moss *L. aduncum* rhizosphere soil), with lower amounts in the MORS (moss *L. aduncum* + *O. hainanensis* rhizosphere soil). The concentration of AK was also slightly higher in the ORS (*O. hainanensis* rhizosphere soil) compared to the MORS (moss *L. aduncum* + *O. hainanensis* rhizosphere soil). OM and TC showed their highest level in the MORS (moss *L. aduncum* + *O. hainanensis* rhizosphere soil), as this condition leads to better organic matter accumulation between these two plant species.

**Figure 3:**
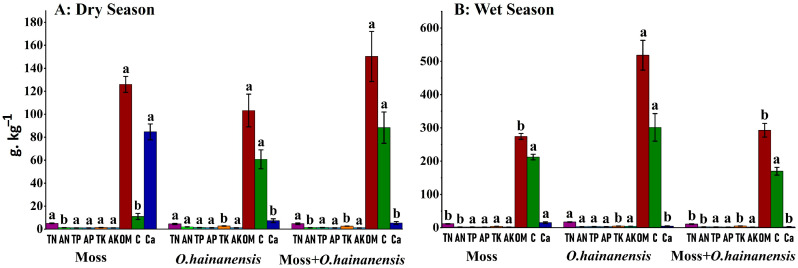
Rhizosphere soil physicochemical parameters among the three groups in the dry and wet seasons. g·kg^−1^. AN: available nitrogen, TN: total nitrogen, OM: organic matter, C: total carbon, AP: available phosphorus, AK: available potassium, Ca^2+^: calcium ion, TP: total phosphorus, and TK: total potassium. The different superscript letters indicate significant differences at *p* < 0.05. All the nutrient values were converted from their measured units (mg/kg, %) to g·kg^−1^ for visual comparison.

In the wet season, several changes were observed in the rhizosphere soil properties among the different plant groups. The TN was highest in the ORS, followed by the MRS, while the MORS had the lowest TN. The concentration of AN was also highest in the ORS, while the MORS and the MRS showed lower levels, reinforcing the role of *O. hainanensis* in promoting nitrogen availability under wet conditions. The concentration of TP was highest in the MORS group. The ORS maintained the highest water content at 191.42%, while the MORS, alongside the MRS, had 78.1% and 74%, respectively, indicating improved water retention of *O. hainanensis* throughout the wet season. Several distinct variations become apparent between dry and wet seasons. Overall, during the wet season, all plant groups demonstrate improved nutrient availability together with higher organic matter content and better soil moisture, which leads to *O. hainanensis* showing significant changes in nitrogen and phosphorus cycling dynamics (Table S2; Figure 3).

Moreover, the root physicochemical parameters across the three groups—single plant group MR–moss *L. aduncum* root, *OHR-O. hainanensis root,* and the combined plant sample MOR–moss *L. aduncum* + *O. hainanensis* root exhibits variability during the dry season. Regarding N accumulation, the MOR showed the significantly highest nitrogen concentration, representing a substantial nitrogen accumulation and facilitative interaction among co-occurring plants. The OHR also displayed a relatively high nitrogen level at 15 g·kg^−1^, while the MR had the lowest nitrogen content, at 4.7 g·kg^−1^ P levels, following a similar trend to the OHR, containing the highest phosphorus content, while the MOR had a slightly lower content. The level of K was also highest in the OHR, while the MR had the lowest level.

**Figure 4:**
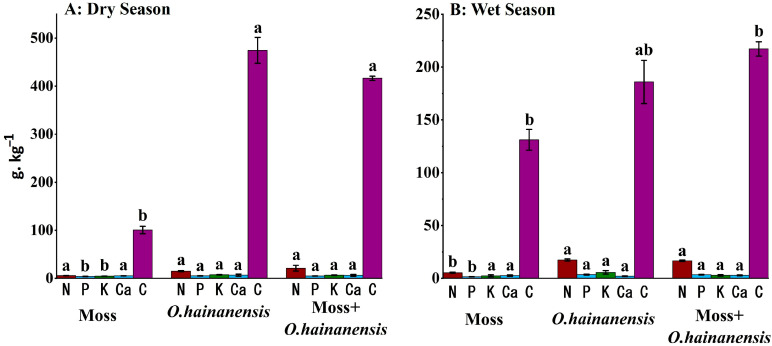
Root physicochemical parameters among the different groups, both in the dry and wet seasons. g·kg^−1^. N: nitrogen; C: total carbon; P: phosphorus; Ca: calcium; and K: potassium. All the nutrient values were converted from their measured units (mg/kg, %) to g·kg^−1^ for visual comparison. The different superscript letters indicate significant differences at *p* < 0.05.

The wet season brings significant changes to root physicochemical parameter patterns when compared to the dry season. N remained the highest in the OHR. The N levels in the MOR showed a slight decrease to 16.61 g·kg^−1^, while the MR group exhibited a slight rise in nitrogen content to 5.33 g·kg^−1^ during the wet season compared to the dry season, representing enhanced nitrogen content in the wet season. The P levels remain highest in the OHR, with the MOR showing slightly lower values. The phosphorus content in the MR increased over the dry season, demonstrating that wetter conditions likely improve phosphorus accessibility for moss *L. aduncum*. The K levels were highest in the OHR, slightly higher than in the dry season, while the MOR had a moderate K level. The TC content was higher in the MOR, but there was a notable decrease compared to the dry season, while the MR experienced an increase in TC levels compared to the dry season. Overall, these contrasts highlight the intricate interactions between plant type and seasonal moisture, manipulating the nutrient availability (Table S3; Figure 4).

### 2.2. Soil, Rhizosphere Soil, and Root PCA

**Figure 5:**
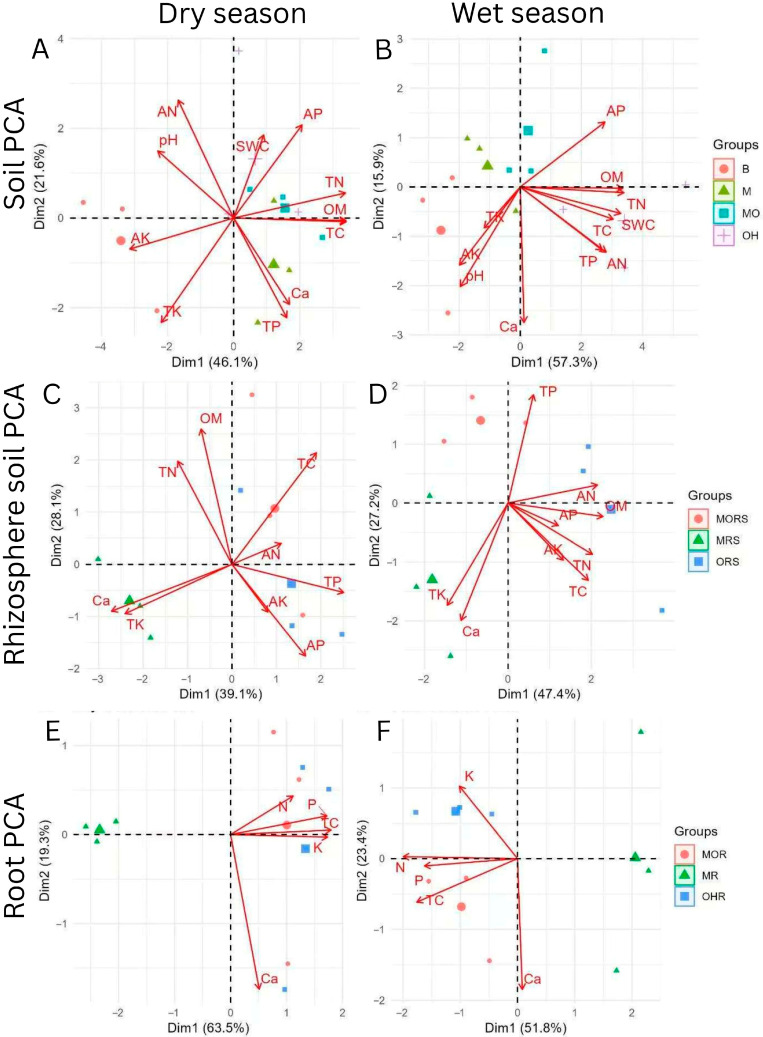
The soil PCA (**A**,**B**), rhizosphere soil PCA (**C**,**D**), and root PCA (**E**,**F**) among the different groups during the dry and wet seasons. B: bare soil, M: moss *L. aduncum* soil, OH: *O. hainanensis* soil, and MO: moss *L. aduncum* + *O. hainanensis* soil. MRS: moss *L. aduncum* rhizosphere soil, MORS: moss *L. aduncum* + *O. hainanensis* rhizosphere soil, and ORS: *O. hainanensis* rhizosphere soil. MR: moss *L. aduncum* root, OHR: *O. hainanensis* root, and MOR: moss *L. aduncum* + *O. hainanensis* root. AN: available nitrogen, TN: total nitrogen, OM: organic matter, TC: total carbon, AP: available phosphorus, AK: available potassium, Ca^2+^: calcium ion, TP: total phosphorus, and TK: total potassium. On the X and Y axes, the PC percent variation is explained.

During the dry season for the soil, PC1 showed a 46.1% variation, more than PC2, at 21.6%, with a total of 67.7%. The single plant sample OH and the combined plant sample MO were clustered near the TN, AP, TC, and OM. In contrast, sample B was more dispersed, showing the low availability of nutrients and possibly lower microbial activity. This pattern reflects a clear separation between bare and plant-associated samples based on nutrient status. During the wet season, PC1 stands at 57.3% higher than PC2, at 15.9%, with total variation explained at 73.2%, reflecting a higher influence of environmental factors, such as moisture and variables. The TN, AP, OM, TC, and SWC were clustered closely, emphasizing the impact of moisture on nutrient retention, while the AN and TP were more linked to PC1. In summary, the single plant sample OH and the combined plant sample MO had higher nutrient levels among seasons, while the sample B had less. Seasonal changes were accompanied by a more pronounced nutrient–moisture alignment in the wet season, as shown by the PCA (Figure 5A,B).

During the dry season for the rhizosphere soil, PC1 accounted for 39.1% more than PC2, at 28.1%. Samples ORS and MORS clustered near the TP, TC, and OM, showing that these plant-associated rhizosphere samples were enriched in phosphorus and carbon-related nutrients and separated from sample MRS. PC2 shows the TN and OM, suggesting that nitrogen dynamics varied more strongly along this axis. During the wet season, PC1 stands at 47.4% higher than PC2, at 27.2%. PC1 highlights MORS supporting the TP, while sample ORS is associated with OM and AN, showing the shifts in nutrient uptake between the two plant types. PC2 distinguishes the nutrient availability, such as TN and OM, from Ca and TK, showing a complex interplay between nutrient types under higher moisture conditions. In summary, the distribution of nutrients differs due to the seasons, with stronger OM and nitrogen impact in the dry season, while phosphorus and available nutrients dominate during the wet season (Figure 5C,D).

During the dry season for the roots, PC1 stands at 63.5%, greater than PC2, at 19.3% of variance. PC1 separates sample OHR and MOR from the MR, though Ca is negatively correlated. Sample OHR and MOR are closely linked to N, P, and K due to stronger associations with these nutrients. PC2 further discriminates the nutrient influence, as TC and P were slightly aligned along this axis, representing the variability in their effects across the root groups. During the wet season, PC1 stands at 51.8% higher than PC2, at 23.4%. PC1 shows that samples MOR and OHR are overlapping, with K strongly positive and a Ca negative correlation, showing consistent nutrient enrichment in the root. Overall, the clear variation in the nutrient profile is higher in the dry season. However, during the wet season, PC2 shows different nutrient relationships, which could be due to increased nutrient mobility or redistribution of elements under higher soil moisture (Figure 5E,F).

### 2.3. Bacterial Community

To investigate the interactions between moss *L. aduncum* and *O. hainanensis*, we used metagenomics sequencing to analyze the microbial communities and physicochemical parameters during the dry and wet seasons. During the dry season, the bacterial species richness in soil samples B, O, M, and MO maintained a steady pattern among all groups. The highest bacterial richness was observed in the single plant sample O, while the lowest was in sample M. This indicates that the single plant *O. hainanensis* demonstrated moderately greater bacterial richness than the combined plant samples. In the wet season, bacterial richness increased across all sample types compared to the dry season. Here, the combined plant soil sample MO exhibited the highest bacterial richness, while the soil sample M contained the lowest. This increase in bacterial richness in the combined plant group during the wet season may reflect enhanced microbial recruitment in coexisting plant environments. In the rhizosphere soil in the dry season, the single plant sample ORS exhibited the highest bacterial richness, while the single plant sample MRS showed the lowest richness. In the wet season, bacterial richness was highest in the rhizosphere soil of the ORS, closely followed by the MORS. The rhizosphere soil of the MRS group contained a lower richness, similar to the dry season. This indicates that bacterial richness increases in the wet season across all groups, with the highest richness still in ORS under wet conditions. Similarly, during the dry season, the root group OHR had the highest bacterial richness, indicating that this group supports the most diverse bacterial community, and the root group MOR had lower richness. Conversely, in the wet season, the roots in the MOR group exhibited the highest bacterial richness, while the MR group showed the lowest bacterial richness. This indicates that bacterial diversity peaked in the MOR group and was lower in the MR, reflecting distinct microbial structures associated with plants under wet conditions, which may enhance microbial diversity through complementary resource facilitation (Table S4; Figure S1A,C). Overall, during the dry season, the single-plant *O. hainanensis* exhibited higher bacterial richness, though in the wet season, bacterial richness increased in all groups, with the MO plant group displaying the maximal bacterial diversity across soil and root samples, possibly reflecting enhanced microbial recruitment or niche expansion under co-occurrence and moist conditions.

**Figure 6:**
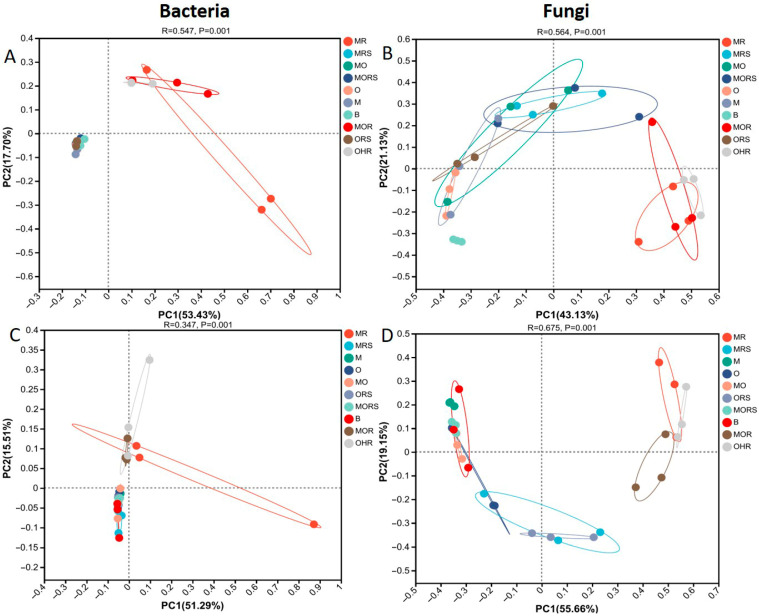
Principal coordinate analysis between microbial communities in soil, rhizosphere soil, and root samples. Bacterial PCoA in dry season (**A**); fungal PCoA in dry season (**B**); bacterial PCoA in wet season (**C**), fungal PCoA in wet season (**D**), OHR: *O. hainanensis* root, MRS: moss *L. aduncum* rhizosphere soil, MOR: moss *L. aduncum* + *O. hainanensis* root, M: moss *L. aduncum* soil, MR: moss *L. aduncum* root, O: *O. hainanensis* soil, ORS: *O. hainanensis* rhizosphere soil, MRS: moss *L. aduncum* rhizosphere soil, MR: moss *L. aduncum* root, MO: moss *L. aduncum* + *O. hainanensis* soil, B: bare limestone, and MORS: moss *L. aduncum* + *O. hainanensis* rhizosphere soil. On the X and Y axes, the PC percent variation is explained, and on the other side, there are the group names, as well as the R and *p* values.

Similarly, in the dry season for the soil groups, sample MO exhibited the highest bacterial Shannon diversity, while sample M had the lowest Shannon diversity. Similarly, in the wet season, sample B had the highest bacterial Shannon diversity, while sample MO contained low Shannon diversity. In the rhizosphere soil in the dry season, sample MRS attained the highest bacterial Shannon diversity. This indicates that sample MRS supports the highest bacterial diversity in the dry season, while sample ORS supports the lowest, though the differences among the groups are relatively small. In the wet season, bacterial Shannon diversity was highest in sample MRS, followed by the rhizosphere soil in sample MORS, and sample ORS had the lowest diversity. These patterns indicate that moss *L. aduncum* roots may provide a more favorable microhabitat for diverse bacterial taxa under both seasonal conditions. During the dry season, the roots in sample MR had the highest Shannon index value, while the roots in sample MOR had the lowest Shannon index. In the wet season, the roots in sample OHR had the highest Shannon diversity, while the roots in sample MOR had the lowest. (Table S4; Figure S2A,C). Overall, the variation in bacterial diversity reflects both plant identity and co-existence effects across the seasons, offering insights into how facilitation or competition may shape microbial community structure. The PCoA analysis revealed that microbial community composition differed significantly among the soil, rhizosphere soil, and roots, with 71.13% and 66.8% explained by microbial community structure in the dry and wet seasons. The first PC1 stands for 53.43% and is higher than PC2, which accounts for 17.70% in the dry season and captures most of the variability. Similarly, in the wet season, PC1 stands for 51.29%, while PC2 accounts for 15.51% (Figure 6A,C). This spatial separation on ordination plots underscores the compartmental and seasonal influence on microbial community dynamics, particularly the distinct structuring between soil-, rhizosphere soil-, and root-associated bacteria.

**Figure 7:**
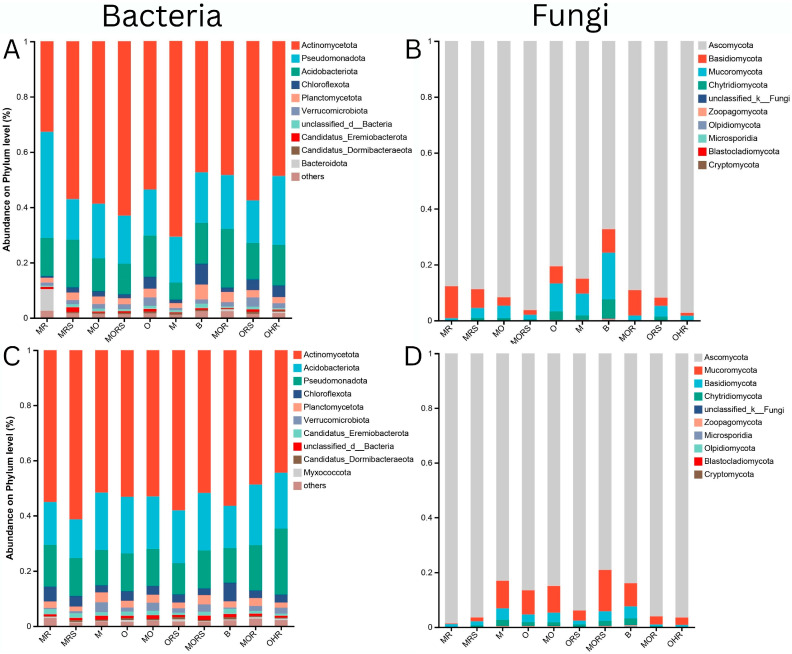
The bacterial and fungal community composition on the phylum level in the dry and wet seasons. Bacteria in the dry season (**A**); fungi in the dry season (**B**); bacteria in the wet season (**C**); and fungi in the wet season (**D**). OHR: *O. hainanensis* root, MRS: moss *L. aduncum* rhizosphere soil, MOR: moss *L. aduncum* + *O. hainanensis* root, M: moss *L. aduncum* soil, MR: moss *L. aduncum* root, O: *O. hainanensis* soil, ORS: *O. hainanensis* rhizosphere soil, MRS: moss *L. aduncum* rhizosphere soil, MR: moss *L. aduncum* root, MO: moss *L. aduncum* + *O. hainanensis* soil, B: bare limestone, and MORS: moss *L. aduncum* + *O. hainanensis* rhizosphere soil.

The bacterial community composition at the phylum level differs across different plant groups and is influenced by seasonal changes. In the dry and wet seasons, the phylum *Actinomycetota* remains the dominant phylum across all groups, representing its resilience to environmental fluctuations and its critical role in sustaining microbial stability and ecological importance. However, differences in the seasons and shifts in the relative abundance of other phyla are notable. In the soil groups (B, M, O, and MO) in the dry season, the microbial composition exhibits a higher proportion of *Actinomycetota*, *Pseudomonadota*, *Acidobacteria*, and *Chloroflexi*. Among them, *Actinomycetota* were relatively higher, particularly in the single plant group M, followed by the combined plant group MO, showing that moss *L. aduncum*-dominated soil may provide conditions that selectively enrich for *Actinomycetota*. The rhizosphere soil groups (MRS, ORS, and MORS) displayed a higher proportion of *Actinomycetota* in the MORS group. The bacterial communities associated with the root groups were also dominated by the phylum *Actinomycetota*, but there was a clear increase in the proportion of *Bacteroidota*, which may specify root-specific microbial relations. In contrast, the wet season discloses considerable shifts in the community composition of bacteria. While *Actinomycetota* remain the dominant phylum, their relative abundance appears reduced among all the groups, particularly in the soil and rhizosphere soil. Conversely, *Acidobacteria* and *Pseudomonadota* exhibit prominent increases, especially in the rhizosphere soil, which could be due to higher levels of moisture enhancing their metabolic activity and niche competitiveness. Additionally, the improved presence of phylum *Myxococcota* in the wet season, which is almost absent in the dry season, could suggest predatory bacterial interactions under wet conditions. Bacterial communities related to soil and roots show a decline in *Bacteroidota*, highlighting the potential seasonal dynamics determined by the nutrient fluxes and seasonal factors (Figure 7). Overall, despite the persistence of a stable core microbiome, seasonal moisture availability appears to be a key driver modulating phylum-level abundances and microbial interactions. The observed differences between soil, rhizosphere soil, and root groups further support the hypothesis that plant–plant interactions influence the microbial communities in response to seasonal changes.

Additionally, the Venn diagram reveals that the genus differed among groups in both the dry and wet seasons. In the dry season, the number of genera ranged from 4 (MO and MORS) to 46 (B). However, a total of 2340 genera were shared between all the groups. Similarly, the number of genera in the wet season ranged from 3 (O and MORS) to 29 (OHR), while a total of 2764 genera were shared among all the groups; these were defined as the core microorganisms of bacterial communities (Figure S3A,C).

Hierarchical cluster analysis demonstrated that samples from the bacterial community were clustered into soil, rhizosphere soil, and root groups that corresponded very well both in the dry and wet seasons. Clustering demonstrated that the bacterial community structure was closely linked to the bryophyte and angiosperm species. So, the results suggested that bryophytes and angiosperms could be a major factor in altering the microbial community differentiation in the rocky ecosystem (Figure S4A,C).

The analysis of LEfSe was used to identify the discriminative bacterial taxon among different groups in both seasons. LEfSe analysis of all groups revealed 645 taxa of bacteria in the dry season and 290 in the wet season, with significant differences (*p* ˂ 0.05, LDA ˃ 2.0) (Figures S5A,C and S6A,C). A multigroup comparison test was further carried out using the Kruskal–Wallis rank test to determine the first 20 genera of bacteria to see the significant differences among the groups, both in the dry and wet seasons. The proportion of *Mycobacterium* was higher in group MR, while lower in OHR, both in the dry and wet seasons. Similarly, *Streptosporangiaceae* was higher in M and lower in MR during the dry season, while in the wet season, it was higher in MRS and lower in MR, respectively (Figures S7A and S8A).

### 2.4. Fungal Community

The alpha diversity in the fungal community among all the groups in both the dry and wet seasons is shown in Table S4. In the dry season, in the soil groups, fungal species richness shows a more pronounced variation, with the combined plant soil sample MO having the highest species richness and sample B having the lowest species richness. This indicates that fungal species richness is higher in the combined plant–soil group. In the wet season, the fungi showed higher richness, with sample MO having the highest species richness and sample B the lowest. For the rhizosphere soil of fungi in the dry season, the MRS and MORS samples had similar richness values, with the MRS sample being slightly higher. The rhizosphere soil in the ORS sample showed the lowest fungal richness. Conversely, in the wet season, sample ORS had the highest fungal richness, followed by the MRS sample, and the MORS sample showed the lowest richness. During the dry season, sample MR showed the highest richness, and sample MOR had slightly lower richness, while sample OHR had the lowest fungal richness. In contrast, during the wet season, the OHR sample showed the highest richness, indicating the greatest fungal diversity, while the sample MOR group had a slightly lower richness (Table S4; Figure S1B,D).

In the dry season for the soil samples, O and MO displayed the highest fungal Shannon diversity, while sample B showed the lowest. Conversely, in the wet season, sample O showed the highest fungal diversity compared to sample MO, while sample M showed the lowest. In contrast, during the dry season in the rhizosphere soil groups, the highest Shannon diversity was found in sample ORS, while sample MORS showed the lowest diversity. In the wet season, sample MORS showed the highest diversity, while sample MRS showed a slightly lower Shannon diversity. The roots in the sample MR group also showed the highest Shannon index value, indicating the greatest fungal diversity, and the MOR group showed the lowest. During the wet season, the MOR group had the highest Shannon index value, while sample MR showed the lowest (Table S4; Figure S2B,D).

The PCoA analysis unveiled a 64.26% variation in fungal community structure in the dry season. The PC1 stands for 43.13% and is higher than PC2, which stands for 21.13%. However, the wet season unveiled 74.81% of the total variation. PC1 stands for 55.66%, while PC2 is 19.15% (Figure 6B,D).

Fungal community composition at the phylum level shows a distinct structure compared to the bacterial communities and displays clear seasonal variation. Across all groups, *Ascomycota* is the most dominant fungal phylum in both the dry and wet seasons. During the dry season, fungal communities in the soil groups (B, M, O, and MO) were highly dominated by the phylum *Ascomycota*, with minimal demonstration of other fungal phyla. *Basidiomycota* and *Chytridiomycota* appeared in lower proportions, mainly in rhizosphere-associated groups (MRS, ORS, and MORS). Moreover, root-associated (MR, OHR, and MOR) fungal communities also unveiled high dominance of *Ascomycota*, a slightly higher proportion of *Mucoromycota,* and *Chytridiomycota* was observed, suggesting that root-associated fungi might include some functionally important taxa involved in the symbiosis or the decomposition processes. The wet season discloses a significant increase in the fungal diversity, with greater demonstration of *Basidiomycota*, *Mucoromycota*, and *Chytridiomycota* across all the groups. The rhizosphere soil groups (MRS, ORS, and MORS) showed a noticeable increase in the phylum *Basidiomycota*, signifying potential shifts in the fungal functional roles in response to increased moisture. *Chytridiomycota* appeared more abundant in wet-season root-associated samples (MR, OHR, and MOR), proposing enhanced fungal activity under wet conditions. In summary, while *Ascomycota* remains the dominant phylum across all groups, fungal community composition is more dynamic than bacterial communities, with seasonal shifts favoring the increased representation of *Basidiomycota*, *Chytridiomycota*, and *Mucoromycota* in the wet season (Figure 7B,D).

The Venn diagram reveals that the genera differed between all groups in the dry and wet seasons. The number ranged from 0 (MRS, MORS, B, and ORS) to 82 (MR). Moreover, a total of 262 were shared between all groups in the dry season. Similarly, in the wet season, the number ranged from 0 (M and ORS) to 15 (MR), while the total number of shared genera among all groups was 242. These were defined as the fungal community core microorganisms (Figure S3B,D).

The analysis of the hierarchical cluster demonstrated that the samples of fungal community from both seasons that were clustered into soil, rhizosphere soil, and root groups corresponded very well. The clustering indicated that the structure of the fungal community was closely linked to the bryophyte and angiosperm species. So, the results suggested that bryophytes and angiosperms could be a major factor in altering the fungal community differentiation in the rocky ecosystem (Figure S4B,D). LEfSe analysis of all groups revealed 222 taxa of fungi in the dry season and 290 in the wet season with significant differences (*p* ˂ 0.05, LDA ˃ 2.0) (Figures S5B,D and S6B,D). Furthermore, a multigroup comparison using the Kruskal–Wallis rank test was further carried out to determine the first 20 genera of fungi to see the significant differences among the groups, both in the dry and wet seasons. The proportion of *Aspergillus* was higher in the M group during the wet season and the B group during the dry season, while lower in the MR group in both the dry and wet seasons. *Rhizopus* was higher in the M group and lower in the MR group during the wet season, while higher in group B and lower in the MOR group during the dry season (Figures S7B and S8B).

### 2.5. Relationship Between Microbial Communities and Soil, Rhizosphere Soil, and Root Variables

**Figure 8:**
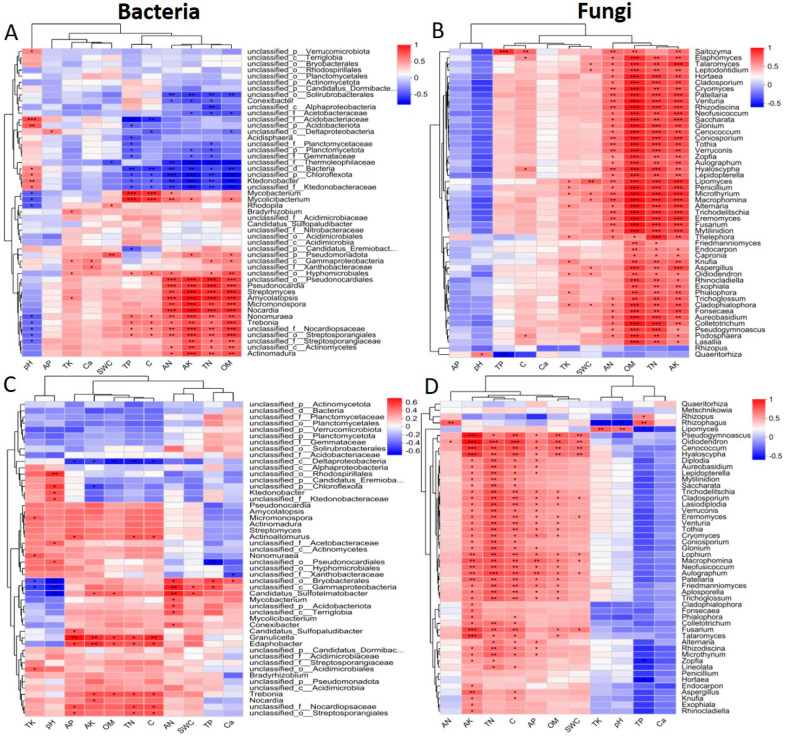
Association of bacteria and fungi with the soil and rhizosphere soil variables in the dry and wet seasons. Bacteria in the dry season (**A**); fungi in the dry season (**B**); bacteria in the wet season (**C**); and fungi in the wet season (**D**). The top 50 abundant genera were selected to calculate the Spearman’s correlation values for each variable. The asterisks (*, **, and ***) show significant correlation at *p*-value < 0.05, 0.01, and 0.001. AN: available nitrogen, TN: total nitrogen, OM: organic matter, C: total carbon, AP: available phosphorus, AK: available potassium, Ca^2+^: calcium ion, TP: total phosphorus, TK: total potassium, and SWC: soil water content.

**Figure 9:**
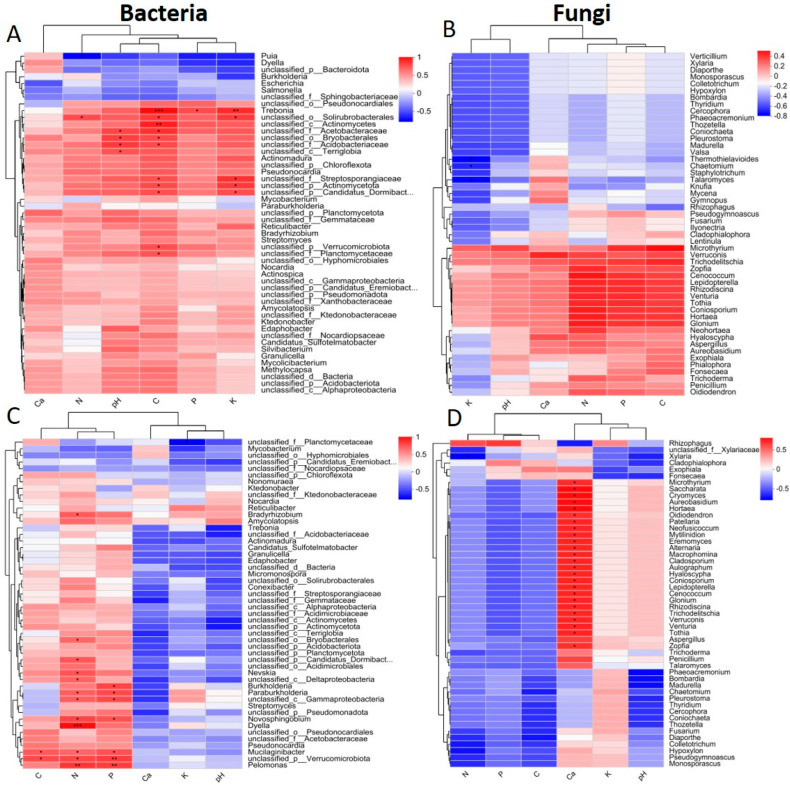
Bacterial and fungal association with root variables in both the dry and wet seasons. A: bacteria + variables in dry season bacteria (**A**); fungi + variables in dry season fungi (**B**); bacteria + variables in wet season (**C**); and fungi + variables in wet season (**D**). The top 50 abundant genera were selected to calculate the Spearman’s correlation values for each variable. The asterisks (*, **, and ***) show significant correlation at *p*-value < 0.05, 0.01, and 0.001. N: nitrogen, C: carbon, P: phosphorus, Ca^2+^: calcium, and K: potassium.

We further demonstrated the correlation between the specific genera in the microbial community and the soil, rhizosphere, and root traits of bare, moss *L. aduncum*, *O. hainanensis*, and moss *L. aduncum* + *O. hainanensis* in both the dry and wet seasons using the Spearman rank correlation analysis. The top 50 most abundant genera were chosen for the analysis of the bacterial and fungal communities based on a systematic perspective. According to the LEfSe analysis, the main microbiota that was differentially abundant was used to construct the values of Spearman’s rank correlation for each genus. The results of the Spearman’s rank correlation analysis revealed clear clustering patterns in the bacteria and fungi. For instance, the stronger correlation between N, P, K, AN, AP, AK, OM, and C with bacterial genera, such as *Trebonia*, *Streptomyces*, *Pseudonocardia*, *Amycolatopsis*, *Nonomuraea*, *Actinomadura*, *Mycobacterium*, *Nocardia*, *Actinomundra*, *Pelamonas*, and *Bradyrhizobium.* In parallel, a positive correlation was noted between soil, rhizosphere soil, root variables, and the top 50 fungal genera (Figure 8 and Figure 9). The results underscore the functional relevance of microbial assemblages in supporting bryophyte and angiosperm co-existence on limestone.

### 2.6. Microbial Functional Analysis

**Figure 10:**
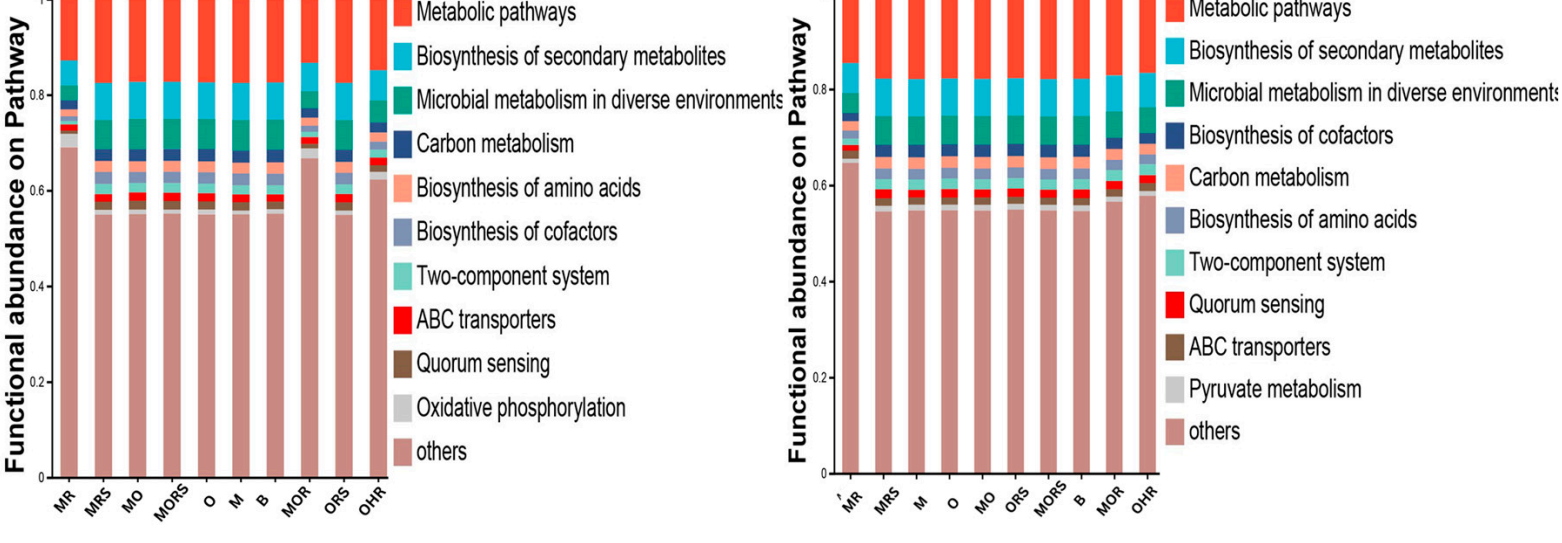
Relative abundance of dominant functional KEGG pathways during dry and wet seasons.

To explore the functional pathways of microbial communities, we performed the functional annotation of microorganisms based on the KEGG database to compare the relative abundance in both the dry and wet seasons. Metabolic pathways were the most dominant pathways across all the samples, followed by biosynthesis of secondary metabolites, microbial metabolism in diverse environments, and carbon metabolism. ABC transporters were also observed in both the dry and wet seasons. The results underscore how functional traits of microbial communities mirror the ecological pressure imposed by the harsh environment (Figure 10).

## 3. Discussion

Microbes are regarded as the second set of genomes in plants and play a vital role in nutrient recycling, promoting the growth of plants, development, and inhibiting fungal pathogens [16]. The microbial community richness and diversity altered dramatically among the groups according to the alpha diversity results in both the dry and wet seasons, which is consistent with the previously published study by Hong et al. [17]. These shifts in microbial diversity and composition suggest that different plant combinations influence microbial colonization and diversity in ways that may modify nutrient turnover and plant growth strategies. For instance, the consistently higher bacterial richness observed in the rhizosphere and root zones suggests that certain plant species may enhance microbial colonization, possibly through the secretion of root exudates, altered root architecture, or physiological traits that selectively recruit beneficial microbes, thereby shaping the structure and function of the root-associated microbiome, as previously reported in a study by Mendes et al. [18]. Generally, linked with the habitat type of limestone, the species of bryophyte and angiosperm play a significant role in shaping the microbial community structure, as supported by the observed clustering patterns and LEfSe analysis, which is consistent with the previous findings, as plant identity and habitat type influence the microbial community composition, as reported by Delgado-Baquerizo et al. [19]. The dominance of different microbial taxa under single and combined plant groups suggests distinct belowground microbial assemblages, which may reflect species-specific root traits, nutrient acquisition strategies, or microhabitat modifications. These differences suggest that plant identity can mediate plant–plant interactions indirectly through their influence on the soil microbial community, as stated by Van Der Heijden et al. [20]. According to our findings, nitrogen dynamics suggest shifts in resource use strategies between species across different environmental conditions. The AN and TN concentrations in the soil and rhizosphere soil in the combined plant groups MO and MORS decreased against the single plant group, which may indicate the intensified resource uptake, potentially associated with competition during the dry season. Bever et al. stated that the co-occurrence of plant species may enhance nutrient foraging efficiency or microbial cycling, consistent with facilitation under certain seasonal conditions [21]. However, this pattern alone does not confirm competition, as it may also result from differing root traits and species-specific uptake rates. In contrast, the combined plant MOR group displayed significantly higher nitrogen content in the dry season, suggesting potential synergistic effects or enhanced microbial nutrient processing. Microbial collaboration seems to enhance nitrogen acquisition, possibly through the mutual associations between the nitrogen-fixing bacteria together with the fungal endophytes, as demonstrated in a previous study by Devi et al. [22]. Microorganisms play a key role in plant interactions by influencing the nutrient cycling and resource acquisition, highlighting their role in belowground dynamics, as demonstrated in the previously published study by Keymer and Lankau [23]. In this study, the microbial community showed the presence of *Actinomycetota* and *Pseudomonadota* in the combined plant MOR group, which was likely linked to the higher nitrogen content observed in this group, as these taxa play an important role in nitrogen mineralization and organic matter decomposition, as reported by Sepp et al. [24]. This may point to facilitation, where microbial activity increases nitrogen availability, indirectly benefiting co-existing plant species, though causality cannot be confirmed without direct measures of plant performance. Previous research published by Tanvir et al. demonstrated that *Actinomycetota* help to improve the growth of plants by releasing microbial metabolites and supplying nitrogen [25]. Moreover, *Pseudomonadota* plays a major role in nitrogen fixation, plant health, plant biomass, chlorophyll content, and seed germination, as previously reported by Khomutovska et al. [26]. Thus, the abundance of these microbial taxa suggests a possible microbial-mediated facilitative mechanism, where both plant species benefit through improved nutrient availability. This was further supported by the root PCA results, as the MOR was more closely linked to N, indicating that the combined plant group may increase uptake capacity, aligning with the previous study, which shows that root traits and plant–microbe association could enhance nutrient acquisition under limited resources [27]. However, it is important to note that these observations are correlational, while the microbial presence and nutrient levels suggest potential interactions. On the other hand, the rainy season brought increased competition for nitrogen as the MOR group experienced nitrogen deficiency, which may be linked to the enhanced resource demand made possible by favorable moisture conditions for nutrient absorption by the roots [28]. *O. hainanensis* contained higher nitrogen across the soil, rhizosphere, and roots, which may contribute to its dominance and competitive advantage in nitrogen uptake under moist conditions. The microbial patterns displayed similar patterns to the *O. hainanensis* pattern of sustaining *Acidobacteriota* and *Pseudomonadota*, taxa known for their role in nitrogen cycling and nutrient mobilization [29], whereas the MOR group showed an abundance of *Actinomycetota*. Although *Actinomycetota* are commonly associated with nitrogen acquisition and mineralization, their role in the MOR group during the wet season may appear linked to slower nitrogen turnover, potentially immobilizing nitrogen in organic forms and further reducing its availability for plants. This shift may result from a competitive nutrient environment, where *Actinomycetota* contribute to nitrogen immobilization, limiting plant nutrient uptake, and reinforcing competitive disadvantage in the combined plant group [20]. Moreover, different genera of *Actinomycetota* may perform contrasting roles depending on environmental context, such as carbon availability or moisture availability [30,31]. Additionally, the increased presence of quorum sensing and two-component systems during the wet season in the MOR group suggests enhanced microbial signaling, which may further facilitate the competitive interaction by enabling microbes to coordinate stress responses and optimize resource acquisition strategies [32]. This enhanced signaling activity may contribute to reduced nitrogen availability for plants, as microbial communities become more efficient in nutrient sequestration under moist conditions. Such microbial signaling may reflect a community-level strategy favoring dominant species like *O. hainanensis*, supporting coexistence potential. Previous research has indicated that competition predominates in wet habitats because microbial transformation impacts competition rates, whereas facilitation leads to nutrient sharing under certain drought conditions in response to resource limitation [21]. Seasonal differences were similar for the phosphorus, as the combined plant group (MO and MORS) showed higher AP and TP concentrations, which may suggest microbial-influenced changes in phosphorus availability that potentially support coexistence when compared to the single plant group. The elevated phosphorus levels could also be a result of differences in plant uptake efficiency, root foraging behavior, or slower nutrient use, rather than active facilitation [33], or may demonstrate that phosphate-solubilizing bacteria, specifically *Pseudomonadota* and *Actinomycetota*, could also be responsible for the facilitative effect [34]. A previously published study demonstrated that *Actinomycetota* assist in the growth of plants by increasing the availability of phosphorus, further supporting their role in the facilitative effects observed in the combined plant group [35]. Additionally, phosphate-solubilizing bacteria, such as *Bacillus*, *Rhizobium*, and *Enterobacter*, can solubilize the inorganic phosphate. Interestingly, these groups were also observed in the MO and MORS, likely improving the availability of phosphorus for the plants [36]. Moreover, *Acidobacteria*, also present in the MO and MORS group, are recognized for their role in phosphorus solubilization, converting insoluble forms into accessible forms for plants, suggesting a complementary role in sustaining phosphorus availability, especially in the context of the facilitation observed in the combined plant group [37]. According to the PCA results, TP showed a higher interaction with MORS during the wet season (Figure 5). This suggests that its multidimensional involvement in the portioning of nutrient and seasonal dynamics is more pronounced in the rhizosphere soil during the wet season, possibly due to the increased phosphorus mobilization and microbial activity under higher moisture conditions [38]. Nonetheless, further studies including direct plant performance measures would be necessary to confirm whether these microbial and nutrient changes translate into true facilitative or competitive outcomes.

The high phosphorus concentrations in the combined plant roots, comparable to the *O. hainanensis* group, further support the idea that microbes may help plants obtain phosphorus. However, the combined plant group MORS exhibited lower AP values than *O. hainanensis* throughout the wet season in the rhizosphere environments, which could suggest a competitive phosphorus reduction under moisture conditions, reflecting a nutrient uptake advantage by *O. hainanensis*. The fungal community reacted by increasing *Basidiomycota* and *Chytridiomycota* in the combined plant MORS group, suggesting a fungal response to changing availability. These groups are known to mobilize phosphorus through organic matter degradation [39]. This shift may reflect a compensatory microbial strategy to mitigate phosphorus limitation; however, the magnitude of these changes appears insufficient to offset competitive pressure and restore adequate phosphate uptake [40]. *Basidiomycota* is recognized to break down organic phosphorus, thereby making phosphorus more accessible to plants [41]. This implies that under phosphorus-limited and competitive conditions, fungal taxa attempt to buffer nutrient stress, but their facilitative effect may be constrained by plant-level competition for available phosphorus. Moreover, the seasonal variation indicates that microbial phosphorus cycling may play a role in belowground nutrient dynamics, although the direction of interaction cannot be conclusively determined from correlational data alone. This seasonal change highlights that competitive nutrient depletion may dominate in wet conditions, whereas facilitative microbial cycling may become more effective, which becomes active during the dry season when plant nutrient demand is lower [20]. The potassium data further support the evidence that plant–microbe–soil interactions shift seasonally, although the directionality of these interactions remains complex. The combined plant group decreased the potassium level in the soil and rhizosphere soil, including the AK and TK in both seasons, suggesting a possible intensification of potassium uptake. The abundance of *Acidobacteriota*, taxa commonly linked with nutrient-poor environments, in the combined plant group MO and MORS, supports their potential role in mediating these interactions for resources, possibly through oligotrophic strategies that limit rapid resource acquisition by competing species [42]. Sample MOR revealed moderate K levels that showed less potassium than *O. hainanensis,* although competition and facilitation appeared to be balanced between each other, but did not result in higher potassium. However, the roots in *O. hainanensis* (OHR) attained the highest potassium concentrations because of aggressive uptake mechanisms likely facilitated by the potassium-solubilizing bacterial members, such as *Pseudomonas*, *Burkholderia*, which were observed in our study. The study of Sharma et al. reported that these bacterial genera can enhance potassium availability through the solubilization of insoluble forms [43]. Seasonal interactions between plants and microbes can also be understood through carbon and organic matter. The combined plant group MO and MORS reached the highest total carbon and organic matter levels during the dry season, suggesting that microbial decomposition contributed to organic matter accumulation, potentially facilitating the nutrient availability. The combined plant group MO and MORS contained microbial communities that exhibit *Actinomycetota* and *Pseudomonadota*, both of which help in the turnover of organic matter and carbon cycling, thus pointing towards microbial-mediated facilitative process [24,44]. The findings of the PCA further supported the idea of facilitation, as the OM and TC were closely linked to the combined plant group MO; however, in the MORS, only TC was associated, suggesting that facilitative impact may increase the OM and TC. At the same time, the TC has a higher impact on the rhizosphere soil, possibly due to differences in how root-associated microbes and root exudates impact carbon processing and accumulation [45]. However, these associations are correlative and do not establish a direct causal link between root traits and carbon cycling. In the rainy season, the dynamics of the carbon shifted to competition. The level of TC and OM in sample MO and MORS decreased to intermediate, and neither species dominated. This reduction may indicate increased microbial turnover, or shared resources use, rather than direct competition and other mechanisms, such as changes in microbial respiration or plant-specific carbon exudation patterns. While these impacts appeared to be limited, microbial adaptations occurred with higher *Mucoromycota* in the combined plant MORS rhizosphere soil, potentially facilitating nutrient exchange by forming symbiotic associations that reduce competitive stress and enhance phosphorus and carbon uptake under moist conditions [46]. Previously published research has indicated that the bacterial and fungal communities demonstrate a dynamic microbial balancing to maintain nutrient turnover while ameliorating competitive interactions [47]. The roots with dominant *Ascomycota* fungal communities probably obtained nutrients through decomposition mechanisms, which may reflect an adaptation to competitive pressure in a resource-limited environment. This functional capacity of *Ascomycota* supports the idea that microbial shifts underlie plants’ nutrient access strategy, especially when facilitation is weakened by seasonal shifts [22]. Soil water content (SWC), as an indicator of water availability, also modulated the interactions. The *O. hainanensis* soil and rhizosphere soil had the highest SWC content in both the dry and wet seasons, probably due to their deeper root system and large inputs of organic materials, respectively. The lower SWC in the combined group compared to *O. hainanensis* may be a result of overlapping water intake or altered microhabitat structure. This reduced water availability in the combined plant groups may intensify belowground competition, yet microbial mediation can offset some of the competitive stress. Moisture availability is an important factor to promote fungal richness, as wet conditions support the fungal diversity [48]. Therefore, changes in microbial community composition likely influence how plant-to-plant interactions shift between competition and facilitation under various water conditions. Although we observed a pattern where competition appeared more prominent in the wet season and facilitation in the dry season, these conclusions are based on nutrient and microbial correlations, not direct experimental evidence. *Ascomycota’s* common domination in both seasons in the combined plant group suggests that this fungal phylum maintains essential roles in symbiotic interactions, thereby supporting basic trophic functioning in ecosystems [39]. Moreover, microbial communities appear to adapt their composition and function in response to environmental conditions, facilitating nutrient acquisition, enhancing biochemical cycling, and enabling the coexistence of different plant species [20].

While this study frames belowground interactions within a facilitation–competition model, it is important to recognize that this binary framework may oversimplify the nuanced ecological processes operating in a nutrient-poor environment. Plant–plant–microbe interactions often occur along a continuum rather than as discrete categories, and the outcomes can vary with context, spatial scale, and species identity [49]. The stress gradient hypothesis (SGH) may provide a more flexible lens for interpreting seasonal variation in belowground interactions. According to the SGH, facilitative interactions are expected to increase under higher stress, while competition may intensify under more favorable conditions [49]. Our findings also show facilitative interaction during the dry season and increased competition under wet conditions. However, distinguishing true facilitation from coincidental co-occurrence or resource sharing remains challenging without a manipulation experiment. Integrating additional approaches, such as stable isotope tracing and gene expression, could clarify the directionality and plant–microbe–plant interactions [50]. In addition, the incorporation of resource partitioning and temporal niche differentiation may explain how coexisting species reduce direct competition by utilizing different nutrients [51].

### 3.1. Correlations Between Microbes and Physiochemical Traits

Furthermore, the outcomes of Spearman’s rank correlation analysis indicated that microbial communities exhibited a significant correlation with soil, rhizosphere soil, and root variables. The essential nutrients exhibited direct positive correlations with critical bacterial genera, which perform nutrient cycling functions, indicating that microbial processes are closely linked with nutrient availability in plant-associated environments. According to our study, *Streptomyces*—a decomposing genus that produces secondary metabolites [52]—showed positive correlations with organic matter (OM), suggesting its functional role in microbial decomposition and nutrient cycling rather than establishing a direct facilitative effect. Similarly, *Actinomadura* indicates a positive correlation with nitrogen across both seasons, which may reflect its involvement in nitrogen turnover processes [53]. Additionally, *Burkholderia*, a nitrogen-fixing and phosphorus-solubilizing genus [54], showed a positive correlation with nitrogen and phosphorus, highlighting its ecological role in nutrient transformation. While these patterns suggest microbial contributions to nutrient availability, the observed correlations do not confirm that facilitation or competition between plant species is microbial-driven. These correlations are indicative of co-occurring trends and point towards possible microbial mediation in nutrient access. Therefore, interpretations regarding plant–plant interactions made with caution, acknowledging that these microbial correlations might also be influenced by factors such as plant-specific root traits, metabolic profiles, or soil properties [55]. Nevertheless, these microbial–nutrient associations may contribute to shaping the biotic environment that modulates interactions between coexisting plant species under limestone conditions.

### 3.2. KEGG Functional Pathways

Moreover, the KEGG pathway results suggested variations during different seasons that described functional changes in the microbial communities, which may reflect potential shifts in facilitative or competitive interactions among *O. hainanensis* and moss *L. aduncum*. The metabolic pathways, together with the biosynthesis of secondary metabolites and carbon metabolism, were prominent functions across seasons, with differences in relative abundance. These pathway levels suggest that microbial communities are active participants in plant–plant interactions by modulating nutrient dynamics and stress responses. The observed functional adaptations further highlight that microbes adopt distinct strategies for nutrient acquisition and environmental stress tolerance depending on seasonal conditions [44]. The microorganisms showed different ways of adapting to stress and acquiring nutrients during both seasons. The combined plant group MOR showed enhanced oxidative phosphorylation ability along with increased ABC transporters in the dry season, indicating a microbial response to resource limitation, which could be associated with the facilitative interaction because the resource availability, such as water and nutrients, was limited. Such microbial contributions could alleviate nutrient stress and promote plant coexistence, particularly in a nutrient-poor environment [56]. ABC transporters are associated with nutrient uptake and efflux of toxic compounds, potentially benefiting both the plant via improved nutrient availability and detoxification of the shared environment [57]. These findings emphasize the ecological significance of metagenomics functional shifts as a potential driver of belowground plant–plant interaction outcomes. However, this is a correlation-based inference and cannot confirm causality, but only suggests that microbial communities might respond in a way that indirectly supports nutrient sharing under stress. On the other hand, a higher abundance of quorum sensing and two-component systems demonstrates that microbial competition is promoted and microorganisms are potentially more adaptive to nutrient-rich conditions in the wet season [58]. Quorum sensing, in particular, may enhance microbial competition or dominance via coordinated biofilm formation or production of antimicrobial compounds [59], which could indirectly influence plant nutrient access. This observation is a microbial adaptation to intensified plant–microbe or microbe–microbe interactions, possibly contributing to competitive nutrient acquisition. This is in line with the competitive interactions that were noted for the combined plant group in wet conditions, which might reflect intensified microbial activity and communication in nutrient-rich conditions, potentially shaping plant–microbe interactions. Furthermore, comparing the relative stability of carbon metabolism and amino acid biosynthesis in both seasons suggests that the microbial functional stability supports essential ecosystem processes under different plant interactions and seasonal shifts [60]. This functional stability may contribute to maintaining baseline ecosystem processes, such as nutrient cycling, even as competitive pressures vary seasonally. However, the increased enrichment of cofactor biosynthesis during the wet season in our study may suggest active enzymatic processes, probably associated with the utilization of resources and microbial adaptation under a nutrient-enriched environment [61]. Taken together, these functional patterns reflect microbial adjustments to seasonal nutrient and moisture variability, while they suggest possible influences on plant–plant interactions.

## 4. Materials and Methods

### 4.1. Study Site

This study was conducted in Baobaimiao Village in Dongfang city, north of the Changhua River, 18°55′ N, 109°02′ E, elev. Ca. 280 m a.s.l., on bare limestone rocks on Hainan Island, South China, with a mean annual temperature of 22–27 °C and annual precipitation of 1000–2600 mm [62]. The landscape is defined by karst hills and exposed limestone outcrops, underlined by Mesozoic carbonate bedrock, and forms a mosaic environment with shallow, rocky soils interspersed with forest slopes. The vegetation is characteristic of the tropical monsoon rainforest ecoregion, notable for high biodiversity and strong seasonal rainfall patterns marked by evergreen broadleaf forest with high species diversity, dense moss *L. aduncum* mats on limestone surfaces, and the presence of limestone-endemic plants, such as *O. hainanensis* [63]. The soil is characterized by calcareous Leptosols developed from limestone bedrock, featuring a skeletal A horizon rich in coarse limestone fragments over highly permeable limestone substrates, which are poor in water retention due to their minimal thickness (average 4–8 cm in thickness) [64,65]. Field analogues in karst terrains on Hainan report substantial spatial heterogeneity in soil thickness and texture, often with high coarse-fragment content and variable granulometry (sandy loam to loam) [66]. The island is dominated by the summer monsoon and experiences distinct dry (March–May) and rainy (August–October) seasons [62].

### 4.2. Species and Sampling

**Figure 11:**
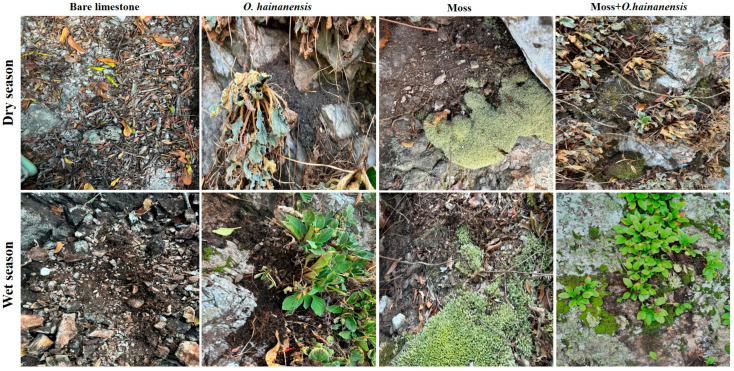
The four different types of groups in the dry and wet seasons: bare limestone, moss *L. aduncum*, *O. hainanensis*, and moss *L. aduncum* + *O. hainanensis*.

In this study, four groups, (I) bare limestone (B), (II) moss *Leucobryum aduncum* (M), (III) *O. hainanensis* (O)*,* and the combined plant group, which refers to the co-occurrence of moss *L. aduncum* and *O. hainanensis*, such as (IV) moss *L. aduncum* + *O. hainanensis* (MO), were included (Figure 11). Three types of samples were collected: soil, rhizosphere soil, and root samples, following established protocols for the compartmentalization of plant-to-soil interfaces [67,68]. The soil was collected from the vicinity of the plants without direct root contact. For the rhizosphere soil of *O. hainanensis*, the plants were gently uprooted, and loosely attached soil was removed by shaking. The soil still adhering to the root surface was carefully brushed off and considered as rhizosphere soil. For the moss *L. aduncum*, rhizosphere soil refers to the soil directly in contact with rhizoids, which were gently separated using sterile brushes. Finally, the root samples of *O. hainanensis* were washed with sterile water to remove the remaining particles and collected for further analysis. For the root samples of moss *L. aduncum*, we collected the rhizoids, and these rhizoids function as their primary substrate attachment structures and were treated as the “root compartment” for comparative purposes. This classification reflects standard practice in microbial ecology to distinguish between soil, rhizosphere soil, and root compartments, as stated in Philippot et al. [55]. A total of 60 samples (30 in dry season and 30 in wet season) of *L. aduncum* (soil and rhizosphere soil), *O. hainanensis* (soil and rhizosphere soil), *L. aduncum* + *O. hainanensis* (soil and rhizosphere soil), and bare limestone (soil) were collected through field sampling. All the samples were divided into two parts, one for the physicochemical properties and the other for the microbial analysis.

### 4.3. Measurement of Soil, Rhizosphere Soil, and Root Variables

The soil and rhizosphere soil variables, including the organic matter (OM), total nitrogen (TN), available nitrogen (AN), total phosphorus (TP), available phosphorus (AP), total potassium (TK), available potassium (AK), pH, soil water content (SWC), calcium ion (Ca^2+^), and total carbon (TC), were analyzed using the standard methods described in the previously published study. The OM and TC were determined by dichromate oxidation using the Walkley–Black method [69], the TN and AN using the Kjeldahl and distillation method [70], the TP and AP by molybdenum blue calorimetry after acid digestion [71], the TK and AK via flame photometry after ammonium acetate extraction [72], the pH with a glass electrode pH meter [73], the SWC by oven drying method, as previously described by Yaseen et al. [74], and Ca^2+^ using the EDTA titration [75]. Root variables, including the nitrogen (N), phosphorus (P), potassium (K), Calcium ion (Ca^2+^), and total carbon (TC), were determined after acid digestion using similar methods, and the pH was measured in homogenized root suspensions. To facilitate the direct comparison across nutrient types in a unified figure, all nutrient concentrations were converted to a common unit of g·kg^−1^ for visualization purposes. The original values were obtained using standard units (i.e., g·kg^−1^, mg/kg, and %) and converted solely for figure presentation, as described previously by Yaseen et al. [74].

### 4.4. Microbial Diversity Analysis

#### 4.4.1. DNA Extraction, Library Construction, and Metagenomics Sequencing

Microbial DNA was extracted from the samples using the Fast DNA^TM^ Spin kit (MP Biomedicals, SC, USA), according to the manufacturer’s protocols. The DNA quality was assessed by 1% agarose gel electrophoresis, and its concentration and purity were measured using the TBS-380 (Turner Biosystems, SC, USA)and Nano-Drop 2000 (Thermo Scientific, WD, USA), respectively. The extracted DNA was fragmented to an average size of about 400 bp using a Covaris M220 (Covaris, WM, USA) (Gene company limited, China) for paired-end library construction. The paired-end library was constructed using NEXTFLEX^®^ Rapid DNA-Sequencing (Bio Scientific, Austin, TX, USA). Adapters comprising the full complement of sequencing primer hybridization sites were ligated to the blunt ends of fragments. Purified amplicons were pooled in equimolar amounts, and paired-end sequencing was accomplished using Illumina Nova Sequencing (Illumina Inc., San Diego, CA, USA) at Majorbio Bio-Pharm Technology Co., Ltd. (Shanghai, China), using Nova Sequencing, 6000 S4 Reagent Kit v1.5 (300 cycles), according to the instructions of the manufacturer [76].

#### 4.4.2. Sequence Quality Control

The data was analyzed on the free online platform, Majorbio Cloud Platform (https://www.majorbio.com/ (accessed on 29 April 2024)). Briefly, the paired-end Illumina reads were trimmed with adaptors, and the reads having low quality (length < 50 bp or with a quality value < 20 or having N bases) were removed by the software fastp (https://github.com/OpenGene/fastp (accessed on 29 April 2024)), version 0.20.0 [77]. The data of metagenomics were assembled using the software MEGAHIT, version 1.1.2 (https://github.com/voutcn/megahit (accessed on 29 April 2024)) [78], which makes use of the succinct de Bruijn graph. Contigs with a length ≥ 300 bp were selected as a result of the final assembly, and then the contigs were used for further prediction and annotation of genes. A catalog of non-redundant genes was built using the software CD-HIT (http://www.bioinformatics.org/cd-hit/), version 4.6.1 (accessed on 29 April 2024) [79], with a sequence identity and coverage of 90%. The high-quality reads were aligned to the catalogs of non-redundant genes to calculate the abundance of genes with 95% identity, using the software SOAP aligner (https://github.com/ShujiaHuang/SOAPaligner, accessed on 29 April 2024) version 2.21 [80]. The demonstrative sequences catalog of non-redundant genes was aligned to the NR database with an e-value cutoff of 1 × 10^−5^, using the software Diamond (https://github.com/bbuchfink/diamond, accessed on 29 April 2024), version 0.8.35, for the taxonomic [81].

### 4.5. Data Analysis

For the analysis of the soil, rhizosphere soil, and root physicochemical parameters, values were represented as the mean ± SD (standard deviation). We used one-way ANOVA followed by Tukey’s HSD test to determine the significant differences (*p* < 0.05) in Statistics 8.1 to evaluate group differences. The bar graph figures were drawn using Origin 2021. The PCA graph figures were drawn using the R software. Alpha diversity measurements, representing the diversity and richness of each sample, were calculated using the R package vegan, version 2.6-4. The relative abundance of bacteria and fungi was assigned at the phylum level based on the metagenomics sequences. The quality of taxonomic assignments is verified by ensuring confidence thresholds and by removing low-quality reads. The dissimilarity of bacterial and fungal communities was determined using principal coordinate analysis (PCoA), based on the Bray–Curtis distance matrices and directed using the vegan package [82]. Shared and unique species among the ten groups were used to create a Venn diagram. Linear discriminant analysis effect size (LEfSe) was performed to investigate potential biomarkers for bacterial and fungal communities among the groups, based on a *p*-value < 0.05 and an LDA score > 2.0 [83]. A multi-group comparison analysis was conducted to assess the interaction among different groups of microbial communities and physicochemical traits using the Kruskal–Wallis rank-sum test (*p*-value ˂ 0.05). Furthermore, the KEGG pathway analysis was conducted by aligning reads to the KEGG database using a BLAST version 2.2.31, search against the Kyoto Encyclopedia of Genes and Genomes (KEGG) database, at an optimized E-value threshold of 1 × 10^−5^.

## 5. Conclusions

To the best of our knowledge, this is the first study to use Illumina Miseq technology for evaluating the facilitation or competition among moss *L. aduncum* and *O. hainanensis* by analyzing microbial community structure and physiochemical attributes associated with the soil, rhizosphere soil, and root on limestone during the dry and wet seasons. In the dry season, the higher nitrogen, phosphorus, carbon, and organic matter levels in the combined plant group may suggest facilitative interactions supported by microbial communities that maintain nutrient cycling in resource-limited environments. Conversely, during the wet season, increased soil moisture leads to more competition, resulting in changes in microbial composition, which may reflect shifts in nutrient uptake strategies rather than direct competition alone, and result in reducing the nutrient levels in the combined plant group. The role of *O. hainanensis* in sustaining the microenvironment in both seasons can be attributed to the elevated soil water content and the rich organic matter, which are conducive to beneficial microbial interactions. In contrast, moss *L. aduncum* has a limited environmental improvement condition due to its low water and nutrient contents. Over the seasons, these variations led microbial communities to respond dynamically, changing their functional potential and composition, which may influence plant coexistence and nutrient cycling. Overall, our work demonstrates that *O. hainanensis* exerts a dominant influence on belowground processes of ecology in limestone ecosystems and that the balance between facilitation and competition is highly context-dependent, seasonal, and mediated by interactions with microbes. However, we acknowledge that the direct measures of plant biomass or growth were not included in this research, which limits the ability to conclusively determine the facilitative or competitive outcomes. Future studies incorporating controlled experiments and plant performance data will be essential to validate the mechanisms driving these interactions.

## Data Availability

The metagenomics data were uploaded to NCBI under Bio Project ID: PRJNA1165716.

## Supplementary Materials

The following supporting information can be downloaded at: https://www.mdpi.com/article/10.3390/plants14162588/s1, Figure S1. Alpha diversity in the bacterial and fungal communities in the dry and wet seasons. The Chao index was calculated at the species level based on the phylogenetic distance. Alpha diversity differences among groups were tested using the Kruskal–Wallis rank test (*p* ˂ 0.05). The bacterial and fungal Chao index in the dry season (A,C) and the bacterial and fungal Chao index in the wet season (B,D). Figure S2. Alpha diversity in the bacterial and fungal communities in dry and wet seasons. The Shannon index was calculated at the species level. Differences in Alpha diversity among groups were tested using the Kruskal–Wallis rank sum test (*p* ˂ 0.05). The bacterial and fungal Shannon index in the dry season (A,C) and the bacterial and fungal Shannon index in the wet season (B,D). Figure S3. Composition similarities and differences of microorganisms in the samples. A, B: Venn diagram of the total and shared bacterial and fungal genera in the dry season (A,B) and the bacterial and fungal total and shared genera in the wet season (C,D). OHR: *O. hainanensis* root, MRS: moss *L. aduncum* rhizosphere soil, MOR: moss *L. aduncum* + *O. hainanensis* root, M: moss *L. aduncum* soil, MR: moss root, O: *O. hainanensis* soil, ORS: *O. hainanensis* rhizosphere soil, MRS: moss *L. aduncum* rhizosphere soil, MR: moss *L. aduncum* root, MO: moss *L. aduncum* + *O. hainanensis* soil, B: bare limestone, and MORS: moss *L. aduncum* + *O. hainanensis* rhizosphere soil. Figure S4. Hierarchical cluster analysis of bacteria and fungi using the Bray–Curtis distance. Bacterial and fungal clustering in the dry season (A,C) and bacterial and fungal clustering in the wet season (B,D). OHR: *O. hainanensis* root, MRS: moss *L. aduncum* rhizosphere soil, MOR: moss *L. aduncum* + *O. hainanensis* root, M: moss *L. aduncum* soil, MR: moss *L. aduncum* root, O: *O. hainanensis* soil, ORS: *O. hainanensis* rhizosphere soil, MRS: moss *L. aduncum* rhizosphere soil, MR: moss *L. aduncum* root, MO: moss *L. aduncum* + *O. hainanensis* soil, B: bare limestone, and MORS: moss *L. aduncum* + *O. hainanensis* rhizosphere soil. Figure S5A–D. The LEfSe results revealed bacterial and fungal biomarkers from the phylum level to the order level that were sensitive to MORS, B, MO, ORS, MR, O, M, MOR, MRS, and OHR in the dry season. There are five circular rings in the cladogram, and each circular ring deposits all taxa within a taxonomic level. The circular ring from inside to outside represents phylum, class, and order, or with an additional family, respectively. The node on the circular ring represents a taxon affiliated with the taxonomic level. Taxa with significantly higher relative abundance in a certain group were color-coded within the cladogram, according to the bacterial and fungal Ribosomal reference (PR2) taxonomy. Figure S6A–D. The LEfSe results revealed bacterial and fungal biomarkers from the phylum level to the order level that were sensitive to MORS, B, MO, ORS, MR, O, M, MOR, MRS, and OHR in the wet season. There are five circular rings in the cladogram, and each circular ring deposits all taxa within a taxonomic level. The circular ring from inside to outside represents phylum, class, and order, or with an additional family, respectively. The node on the circular ring represents a taxon affiliated with the taxonomic level. Taxa with significantly higher relative abundance in a certain group were color-coded within the cladogram, according to the bacterial and fungal Ribosomal reference (PR2) taxonomy. Figure S7A,B. A multigroup comparison test was conducted using the Kruskal–Wallis rank test for the bacterial (A) and fungal (B) groups in the dry season. The bar chart shows the difference in the average relative abundance of the same species between different groups and labels whether the difference was significant (*p*-value, asterisk indicates significant difference). The significance of the differences between the same species in multiple groups is visually demonstrated. The ordinate represents the name of the species at different taxonomic levels, the abscissa represents the percentage value of the abundance of a species in the sample, and different colors represent different groups. 0.01 < *p* ≤ 0.05 *, 0.001 < *p* ≤ 0.01 **, and *p* ≤ 0.001 ***. Figure S8A,B. A multigroup comparison test was conducted using the Kruskal–Wallis rank test for bacterial (A) and fungal (B) groups in the wet season. The bar chart shows the difference in the average relative abundance of the same species between different groups and labels whether the difference was significant (*p*-value, asterisk indicates significant difference). The significance of the differences between the same species in multiple groups is visually demonstrated. The ordinate represents the name of the species at different taxonomic levels, the abscissa represents the percentage value of the abundance of a species in the sample, and different colors represent different groups. 0.01 < *p* ≤ 0.05 *, 0.001 < *p * ≤ 0.01 **, and *p* ≤ 0.001 ***. Table S1. Soil physicochemical traits among four groups in both dry and wet seasons (g·kg^−1^). Table S2. Rhizosphere soil physicochemical traits among three groups in both dry and wet seasons (g·kg^−1^). Table S3. Root physicochemical traits among the three groups in both the dry and wet seasons (g·kg^−1^). Table S4. Richness and diversity indices of bacterial and fungal communities among all groups, both in dry and wet seasons.

## References

[1] Singh A., Shourie A. (2021). Climate Change Impacts on Plant–Microbe Interactions. Climate Change and the Microbiome: Sustenance of the Ecosphere.

[2] Cheng Y.T., Zhang L., He S.Y. (2019). Plant-Microbe Interactions Facing Environmental Challenge. Cell Host Microbe.

[3] Zhang X., Myrold D.D., Shi L., Kuzyakov Y., Dai H., Thu Hoang D.T., Dippold M.A., Meng X., Song X., Li Z. (2021). Resistance of Microbial Community and Its Functional Sensitivity in the Rhizosphere Hotspots to Drought. Soil Biol. Biochem..

[4] Berg G., Rybakova D., Grube M., Köberl M. (2016). The Plant Microbiome Explored: Implications for Experimental Botany. J. Exp. Bot..

[5] Bhattacharyya S.S., Furtak K. (2022). Soil–Plant–Microbe Interactions Determine Soil Biological Fertility by Altering Rhizospheric Nutrient Cycling and Biocrust Formation. Sustainability.

[6] LING S.-J., WEN F., REN M.-X. (2022). Oreocharis Hainanensis (Gesneriaceae), a New Species from Karst Regions in Hainan Island, South China. Phytotaxa.

[7] Gornall J.L., Woodin S.J., Jónsdóttir I.S., van der Wal R. (2011). Balancing Positive and Negative Plant Interactions: How Mosses Structure Vascular Plant Communities. Oecologia.

[8] Sohlberg E.H., Bliss L.C. (1987). Responses of Ranunculus Sabinei and Papaver Radicatum to Removal of the Moss Layer in a High-Arctic Meadow. Can. J. Bot..

[9] Mohan S.M., Sudhakar P. (2022). Metagenomic Approaches for Studying Plant–Microbe Interactions. Understanding the Microbiome Interactions in Agriculture and the Environment.

[10] Bardgett R.D., van der Putten W.H. (2014). Belowground Biodiversity and Ecosystem Functioning. Nature.

[11] Compant S., Clément C., Sessitsch A. (2010). Plant Growth-Promoting Bacteria in the Rhizo- and Endosphere of Plants: Their Role, Colonization, Mechanisms Involved and Prospects for Utilization. Soil Biol. Biochem..

[12] Douda J., Doudová J., Hulík J., Havrdová A., Boublík K. (2018). Reduced Competition Enhances Community Temporal Stability under Conditions of Increasing Environmental Stress. Ecology.

[13] Price T.D., Kirkpatrick M. (2009). Evolutionarily Stable Range Limits Set by Interspecific Competition. Proc. R. Soc. B Biol. Sci..

[14] Cornacchia L., van de Koppel J., van der Wal D., Wharton G., Puijalon S., Bouma T.J. (2018). Landscapes of Facilitation: How Self-organized Patchiness of Aquatic Macrophytes Promotes Diversity in Streams. Ecology.

[15] Gómez-Aparicio L., Zamora R., Castro J., Hódar J.A. (2008). Facilitation of Tree Saplings by Nurse Plants: Microhabitat Amelioration or Protection against Herbivores?. J. Veg. Sci..

[16] Rai A.K., Singh D.P., Prabha R., Kumar M., Sharma L. (2016). Microbial Inoculants: Identification, Characterization, and Applications in the Field. Microbial Inoculants in Sustainable Agricultural Productivity.

[17] Hong C.E., Kim J.U., Lee J.W., Bang K.H., Jo I.H. (2019). Metagenomic Analysis of Bacterial Endophyte Community Structure and Functions in Panax Ginseng at Different Ages. 3 Biotech.

[18] Mendes R., Garbeva P., Raaijmakers J.M. (2013). The Rhizosphere Microbiome: Significance of Plant Beneficial, Plant Pathogenic, and Human Pathogenic Microorganisms. FEMS Microbiol. Rev..

[19] Delgado-Baquerizo M., Reith F., Dennis P.G., Hamonts K., Powell J.R., Young A., Singh B.K., Bissett A. (2018). Ecological Drivers of Soil Microbial Diversity and Soil Biological Networks in the Southern Hemisphere. Ecology.

[20] Van Der Heijden M.G.A., Bardgett R.D., Van Straalen N.M. (2008). The Unseen Majority: Soil Microbes as Drivers of Plant Diversity and Productivity in Terrestrial Ecosystems. Ecol. Lett..

[21] Bever J.D., Dickie I.A., Facelli E., Facelli J.M., Klironomos J., Moora M., Rillig M.C., Stock W.D., Tibbett M., Zobel M. (2010). Rooting Theories of Plant Community Ecology in Microbial Interactions. Trends Ecol. Evol..

[22] Devi R., Kaur T., Kour D., Rana K.L., Yadav A., Yadav A.N. (2020). Beneficial Fungal Communities from Different Habitats and Their Roles in Plant Growth Promotion and Soil Health. Microb. Biosyst..

[23] Keymer D.P., Lankau R.A. (2017). Disruption of Plant–Soil–Microbial Relationships Influences Plant Growth. J. Ecol..

[24] Sepp S.-K., Vasar M., Davison J., Oja J., Anslan S., Al-Quraishy S., Bahram M., Bueno C.G., Cantero J.J., Fabiano E.C. (2023). Global Diversity and Distribution of Nitrogen-Fixing Bacteria in the Soil. Front. Plant Sci..

[25] Tanvir R., Sheikh A.A., Javeed A. (2019). Endophytic Actinomycetes in the Biosynthesis of Bioactive Metabolites: Chemical Diversity and the Role of Medicinal Plants. Stud. Nat. Prod. Chem..

[26] Khomutovska N., Jasser I., Isidorov V.A. (2024). Unraveling the Role of Bacteria in Nitrogen Cycling: Insights from Leaf Litter Decomposition in the Knyszyn Forest. Forests.

[27] Ulrich D.E.M., Sevanto S., Ryan M., Albright M.B.N., Johansen R.B., Dunbar J.M. (2019). Plant-Microbe Interactions before Drought Influence Plant Physiological Responses to Subsequent Severe Drought. Sci. Rep..

[28] Hodge A., Stewart J., Robinson D., Griffiths B.S., Fitter A.H. (2000). Competition between Roots and Soil Micro-organisms for Nutrients from Nitrogen-rich Patches of Varying Complexity. J. Ecol..

[29] Jiao S., Peng Z., Qi J., Gao J., Wei G. (2021). Linking Bacterial-Fungal Relationships to Microbial Diversity and Soil Nutrient Cycling. mSystems.

[30] Jog R., Pandya M., Nareshkumar G., Rajkumar S. (2014). Mechanism of Phosphate Solubilization and Antifungal Activity of Streptomyces Spp. Isolated from Wheat Roots and Rhizosphere and Their Application in Improving Plant Growth. Microbiology.

[31] Hamdali H., Hafidi M., Virolle M.J., Ouhdouch Y. (2008). Rock Phosphate-Solubilizing Actinomycetes: Screening for Plant Growth-Promoting Activities. World J. Microbiol. Biotechnol..

[32] Ng W.-L., Bassler B.L. (2009). Bacterial Quorum-Sensing Network Architectures. Annu. Rev. Genet..

[33] Richardson A.E., Simpson R.J. (2011). Soil Microorganisms Mediating Phosphorus Availability Update on Microbial Phosphorus. Plant Physiol..

[34] Rodríguez H., Fraga R. (1999). Phosphate Solubilizing Bacteria and Their Role in Plant Growth Promotion. Biotechnol. Adv..

[35] Schütze E., Klose M., Merten D., Nietzsche S., Senftleben D., Roth M., Kothe E. (2014). Growth of Streptomycetes in Soil and Their Impact on Bioremediation. J. Hazard. Mater..

[36] Nouioui I., Cortés-albayay C., Carro L., Castro J.F., Gtari M., Ghodhbane-Gtari F., Klenk H.-P., Tisa L.S., Sangal V., Goodfellow M. (2019). Genomic Insights Into Plant-Growth-Promoting Potentialities of the Genus Frankia. Front. Microbiol..

[37] Gonçalves O.S., Fernandes A.S., Tupy S.M., Ferreira T.G., Almeida L.N., Creevey C.J., Santana M.F. (2024). Insights into Plant Interactions and the Biogeochemical Role of the Globally Widespread Acidobacteriota Phylum. Soil Biol. Biochem..

[38] Xu T., Shen Y., Ding Z., Zhu B. (2023). Seasonal Dynamics of Microbial Communities in Rhizosphere and Bulk Soils of Two Temperate Forests. Rhizosphere.

[39] Tedersoo L., Bahram M., Põlme S., Kõljalg U., Yorou N.S., Wijesundera R., Ruiz L.V., Vasco-Palacios A.M., Thu P.Q., Suija A. (2014). Global Diversity and Geography of Soil Fungi. Science.

[40] Jones M.D.M., Forn I., Gadelha C., Egan M.J., Bass D., Massana R., Richards T.A. (2011). Discovery of Novel Intermediate Forms Redefines the Fungal Tree of Life. Nature.

[41] Muneer M.A., Huang X., Hou W., Zhang Y., Cai Y., Munir M.Z., Wu L., Zheng C. (2021). Response of Fungal Diversity, Community Composition, and Functions to Nutrients Management in Red Soil. J. Fungi.

[42] Islam W., Noman A., Naveed H., Huang Z., Chen H.Y.H. (2020). Role of Environmental Factors in Shaping the Soil Microbiome. Environ. Sci. Pollut. Res..

[43] Sharma S.B., Sayyed R.Z., Trivedi M.H., Gobi T.A. (2013). Phosphate Solubilizing Microbes: Sustainable Approach for Managing Phosphorus Deficiency in Agricultural Soils. Springerplus.

[44] Trivedi P., Leach J.E., Tringe S.G., Sa T., Singh B.K. (2020). Plant–Microbiome Interactions: From Community Assembly to Plant Health. Nat. Rev. Microbiol..

[45] Malik A.A., Bouskill N.J. (2022). Drought Impacts on Microbial Trait Distribution and Feedback to Soil Carbon Cycling. Funct. Ecol..

[46] Hodge A., Robinson D., Fitter A. (2000). Are Microorganisms More Effective than Plants at Competing for Nitrogen?. Trends Plant Sci..

[47] Bever J.D., Platt T.G., Morton E.R. (2012). Microbial Population and Community Dynamics on Plant Roots and Their Feedbacks on Plant Communities. Annu. Rev. Microbiol..

[48] Chaparro J.M., Sheflin A.M., Manter D.K., Vivanco J.M. (2012). Manipulating the Soil Microbiome to Increase Soil Health and Plant Fertility. Biol. Fertil. Soils.

[49] Maestre F.T., Callaway R.M., Valladares F., Lortie C.J. (2009). Refining the Stress-gradient Hypothesis for Competition and Facilitation in Plant Communities. J. Ecol..

[50] Aguilar-Trigueros C.A., Hempel S., Powell J.R., Anderson I.C., Antonovics J., Bergmann J., Cavagnaro T.R., Chen B., Hart M.M., Klironomos J. (2015). Branching out: Towards a Trait-Based Understanding of Fungal Ecology. Fungal Biol. Rev..

[51] Chesson P. (2000). Mechanisms of Maintenance of Species Diversity. Annu. Rev. Ecol. Syst..

[52] Kong D., Wang X., Nie J., Niu G. (2019). Regulation of Antibiotic Production by Signaling Molecules in Streptomyces. Front. Microbiol..

[53] Lu C., Zhang Z., Guo P., Wang R., Liu T., Luo J., Hao B., Wang Y., Guo W. (2023). Synergistic Mechanisms of Bioorganic Fertilizer and AMF Driving Rhizosphere Bacterial Community to Improve Phytoremediation Efficiency of Multiple HMs-Contaminated Saline Soil. Sci. Total Environ..

[54] Pang Z., Dong F., Liu Q., Lin W., Hu C., Yuan Z. (2021). Soil Metagenomics Reveals Effects of Continuous Sugarcane Cropping on the Structure and Functional Pathway of Rhizospheric Microbial Community. Front. Microbiol..

[55] Philippot L., Raaijmakers J.M., Lemanceau P., van der Putten W.H. (2013). Going Back to the Roots: The Microbial Ecology of the Rhizosphere. Nat. Rev. Microbiol..

[56] Lemieux H., Blier P.U. (2022). Exploring Thermal Sensitivities and Adaptations of Oxidative Phosphorylation Pathways. Metabolites.

[57] Davidson A.L., Dassa E., Orelle C., Chen J. (2008). Structure, Function, and Evolution of Bacterial ATP-Binding Cassette Systems. Microbiol. Mol. Biol. Rev..

[58] Papenfort K., Bassler B.L. (2016). Quorum Sensing Signal–Response Systems in Gram-Negative Bacteria. Nat. Rev. Microbiol..

[59] Miller M.B., Bassler B.L. (2001). Quorum Sensing in Bacteria. Annu. Rev. Microbiol..

[60] Delgado-Baquerizo M., Maestre F.T., Reich P.B., Jeffries T.C., Gaitan J.J., Encinar D., Berdugo M., Campbell C.D., Singh B.K. (2016). Microbial Diversity Drives Multifunctionality in Terrestrial Ecosystems. Nat. Commun..

[61] Chubukov V., Gerosa L., Kochanowski K., Sauer U. (2014). Coordination of Microbial Metabolism. Nat. Rev. Microbiol..

[62] Yun Q., Zhang Q. (2025). Spatiotemporal Variation Characteristics of Extreme Temperature Events in Hainan Province over the Past Four Decades. Front. Environ. Sci..

[63] Guo J., Zhang L., Qi S., Chen J. (2024). Spatiotemporal Dynamics and Driving Factors of Vegetation Greenness in Typical Tourist Region: A Case Study of Hainan Island, China. Land.

[64] Schad P. (2023). World Reference Base for Soil Resources—Its Fourth Edition and Its History. J. Plant Nutr. Soil Sci..

[65] Chengmin H., Zitong G., Yurong H. (2004). Elemental Geochemistry of a Soil Chronosequence on Basalt on Northern Hainan Island, China. Chin. J. Geochemistry.

[66] Zhang Z., Hu B., Hu G. (2014). Spatial Heterogeneity of Soil Chemical Properties in a Subtropical Karst Forest, Southwest China. Sci. World J..

[67] Edwards J., Johnson C., Santos-Medellín C., Lurie E., Podishetty N.K., Bhatnagar S., Eisen J.A., Sundaresan V. (2015). Structure, Variation, and Assembly of the Root-Associated Microbiomes of Rice. Proc. Natl. Acad. Sci. USA.

[68] Feng J., Xu Y., Ma B., Tang C., Brookes P.C., He Y., Xu J. (2019). Assembly of Root-Associated Microbiomes of Typical Rice Cultivars in Response to Lindane Pollution. Environ. Int..

[69] Walkley A., Black I.A. (1934). An examination of the degtjareff method for determining soil organic matter, and a proposed modification of the chromic acid titration method. Soil Sci..

[70] Bremner J.M. (1965). Total Nitrogen. Methods of Soil Analysi: Part 2 Chemical and Microbiological Properties.

[71] Murphy J., Riley J.P. (1962). A Modified Single Solution Method for the Determination of Phosphate in Natural Waters. Anal. Chim. Acta.

[72] RICHARDS L.A. (1954). Diagnosis and Improvement of Saline and Alkali Soils. Soil Sci..

[73] Peech M. (1965). Hydrogen-Ion Activity. Methods of Soil Analysis: Part 2 Chemical and Microbiological Properties.

[74] Yaseen M., Khan W.R., Bahadur S., Batool F., Khalid F., Ahmed U., Ashraf M. (2023). Intra- and Inter-Specific Responses of Plant Functional Traits to Environmental Variables: Implications for Community Ecology in the Tropical Monsoonal Dwarf Forest on Hainan Island. Front. For. Glob. Change.

[75] Thomas G.W. (1982). Exchangeable Cations. Methods of Soil Analysis: Part 2 Chemical and Microbiological Properties.

[76] Zhong Y., Hu J., Xia Q., Zhang S., Li X., Pan X., Zhao R., Wang R., Yan W., Shangguan Z. (2020). Soil Microbial Mechanisms Promoting Ultrahigh Rice Yield. Soil Biol. Biochem..

[77] Chen S., Zhou Y., Chen Y., Gu J. (2018). Fastp: An Ultra-Fast All-in-One FASTQ Preprocessor. Bioinformatics.

[78] Li D., Liu C.-M., Luo R., Sadakane K., Lam T.-W. (2015). MEGAHIT: An Ultra-Fast Single-Node Solution for Large and Complex Metagenomics Assembly via Succinct de Bruijn Graph. Bioinformatics.

[79] Fu L., Niu B., Zhu Z., Wu S., Li W. (2012). CD-HIT: Accelerated for Clustering the next-Generation Sequencing Data. Bioinformatics.

[80] Li R., Li Y., Kristiansen K., Wang J. (2008). SOAP: Short Oligonucleotide Alignment Program. Bioinformatics.

[81] Buchfink B., Xie C., Huson D.H. (2015). Fast and Sensitive Protein Alignment Using DIAMOND. Nat. Methods.

[82] Liu A., Li Y., Wang Q., Zhang X., Xiong J., Li Y., Lei Y., Sun Y. (2022). Analysis of Microbial Diversity and Community Structure of Rhizosphere Soil of Cistanche Salsa from Different Host Plants. Front. Microbiol..

[83] Segata N., Izard J., Waldron L., Gevers D., Miropolsky L., Garrett W.S., Huttenhower C. (2011). Metagenomic Biomarker Discovery and Explanation. Genome Biol..

