# Supplementary material for: Facilitation in the Dry Season: Species Interactions Between a Limestone-Endemic Plant and Moss Altered by Precipitation Dynamics
Source: Plants (Basel). 2025 Aug 20;14(16):2588. doi: 10.3390/plants14162588 (PMC12389180; doi:10.3390/plants14162588)
Supplement: Supplementary file 1 [file plants-14-02588-s001.zip › plants-3694772-supplementary.pdf]

Table S1. Soil physicochemical traits among four groups in both dry and wet seasons (g/kg).

	Dry Season				Wet Season			
**Variables**	**Bare limestone**	**Moss**	***O. hainanensis***	**Moss + *O. hainanensis***	**Bare limestone**	**Moss**	***O. hainanensis***	**Moss + O. hainanensis**
TN	1±0.25b	3.68±0.63a	3.81±0.80a	3.38±0.87a	4.1±10c	6.55±1.31bc	14.14±2.50a	9.33±0.40b
AN	0.43±0.20a	0.25±0.04a	0.46±0.12a	0.3±0.06a	1±0.21a	0.5±0.11a	1.03±0.33a	1.02±0.10a
TP	0.24±0.05a	0.4±0.1a	0.3±0.10a	0.34±0.03a	0.4±0.13a	0.33±0.2a	0.7±0.20a	0.42±0.03a
AP	0.04±0.01b	0.06±0.03b	0.15±0.05ab	0.2±0.04a	0.05±0.01a	0.11±0.1a	0.3±0.11a	0.22±0.12a
TK	2±0.20a	1.6±0.20ab	1.4±0.14b	1.6±0.10ab	1±0.310a	3.1±0.53a	3±0.31a	2.5±0.25a
AK	1.12±0.14a	0.1±0.05b	0.15±0.10b	0.1±0.10b	2±0.20a	0.2±0.10b	0.22±0.10b	0.13±0.03b
pH	4.2±0.65a	3.7±0.20a	4.1±0.32a	3.75±0.40a	4±0.53a	3.65±0.12a	3.43±0.21a	3.53±0.25a
OM	26.2±7.00b	101.1±26.42a	77.5±24.0ab	105.13±19.13a	88.23±6.70c	189.33±37.6bc	376.33±61.3a	224.33±15b
C	16±4.21b	59.4±15.40a	45.6±14.03ab	62.03±10.21a	64.03±7.40c	109.4±22bc	237.33±25.10a	130.6±18.10b
Ca	4.12±1.41a	5.4±1.25a	5.03±1.66a	5.05±1.63a	2.7±1.002a	2.53±0.50a	3.3±1.54a	1.7±1.11a
SWC	1.4±1.41a	1.04±0.42a	4.7±2.00a	4.21±3.01a	44.31±80b	58.62±8.61b	151.5±23.25a	66.1±9.34b

Here, AN: available nitrogen, TN: total nitrogen, OM: organic matter, C: total carbon, AP: available phosphorus, AK: available potassium, Ca: calcium ion, TP: total phosphorus, TK: total potassium, respectively.

Table S2. Rhizosphere soil physicochemical traits among three groups in both dry and wet seasons (g/kg).

	Dry Season			Wet Season		
**Variables**	**Moss**	***O. hainanensis***	**Moss+*O. hainanensis***	**Moss**	***O. hainanensis***	**Moss+*O. hainanensis***
TN	5.1±0.45a	4.55±10a	5.04±1.20a	12.03±1.11b	17.2±1.40a	11±1.11b
AN	0.24±0.04b	1.04±0.30a	0.3±0.12b	0.4±0.20b	1.3±0.15a	0.75±0.10b
TP	0.12±0.05a	0.32±0.11a	0.35±0.11a	0.24±0.2a	0.5±0.25a	0.51±0.24a
AP	0.1±0.10a	0.2±0.20a	0.11±0.10a	0.2±0.10a	0.3±0.20a	0.21±0.10a
TK	2.4±0.20a	2.03±0.20b	1.63±0.10b	4.1±0.30a	2.8±0.25b	2.9±0.30b
AK	0.12±0.01a	0.15±0.11a	0.11±0.02a	0.3±0.03a	0.3±0.40a	0.24±0.10a
pH	4.34±0.24a	3.83±0.20ab	4.02±0.20b	4.4±0.30a	3.47±0.33b	3.65±0.30b
OM	126±12.02a	103.23±25.03a	150.3±37.7a	274.33±15.4b	518.33±77.6a	293±35.50b
C	11±4.55b	61±14.22a	88.33±24a	212.3±15a	301.33±72.05a	170±20.40a
Ca	85±12	7.4±30	5.4±2.10	15.23±4.51a	5.04±1.23b	2.6±1.20b
SWC	4.11±2a	9.3±40a	3.8±3.50a	74±6b	191.42±27.42a	78.1±11.02b

Here, AN: available nitrogen, TN: total nitrogen, OM: organic matter, C: total carbon, AP: available phosphorus, AK: available potassium, Ca: calcium ion, TP: total phosphorus, TK: total potassium, respectively.

Table S3. Root physicochemical traits among the three groups in both dry and wet seasons (g/kg).

	Dry Season			Wet Season		
**Variables**	**Moss**	***O. hainanensis***	**Moss + *O. hainanensis***	**Moss**	***O. hainanensis***	**Moss + *O. hainanensis***
N	4.7±0.3a	15±2.03a	21.2±10.35a	5.33±1b	17.4±2.03a	16.61±1.2a
P	1.2±0.5b	3.25±0.51a	3.04±0.5a	1.6±0.3b	3.5±1a	3.4±0.5a
K	2.04±0.14b	5.45±1.05a	4.6±1a	2.31±2a	5.61±3a	3.03±1.1a
Ca	2.35±0.3a	5.03±3.5a	4.2±3.15a	2.6±1.1a	2.1±0.45a	3±1.01a
pH	4.7±0.24b	9.01±0.32a	10.01±1a	4.73±0.41a	4.4±0.3a	4.3±1a
C	100.63±13.35b	474.6±46.5a	417±8a	131.1±17.1b	186±35.4ab	217.1±12.04a

Here, N: nitrogen, C: total carbon, P: phosphorus, Ca: calcium, K: potassium, respectively.

Table S4. Richness and diversity indices of bacterial and fungal communities among all groups, both in dry and wet seasons**.**

Seasons	Microbes	Indexes	Root groups			Rhizosphere soil groups			Soil groups				
			**MR**	**OHR**	**MOR**	**MRS**	**ORS**	**MORS**	**B**	**O**	**M**	**MO**	***p*-value**
**Dry Season**	**Bacteria**	**Chao**	10087±4744.1	15219±824.3	13429±1364.4	17674.3±265	18840±866.1	18201.3±245.3	18123.3±193.9	18495.3±354.9	17449.6±287.5	18201.3±496.6	0.004
		**Shannon**	5.15±0.46	4.86±0.26	4.81±0.21	4.34±0.13	4.26±0.11	4.30±0.04	4.14±0.05	4.22±0.05	4.09±0.09	4.29±0.05	0.004
	**Fungi**	**Chao**	1604.6±296.1	1334±42.6	1357.6±179.5	1011.6±154.9	751.6±129.1	1010.6±229.1	316.6±15.6	561.6±80.4	697.3±277.1	802.6±287.7	0.003
		**Shannon**	5.55± 0.31	5.49±0.15	5.27±0.31	5.37± 0.21	5.47±0.08	5.31±0.12	4.61± 0.19	5.32±0.25	5.19± 0.36	5.32± 0.17	0.2
**Wet season**	**Bacteria**	**Chao**	11992±4975.1	210161145.1	21239.3±770.6	17785±1467	20218±272.9	20098±946.8	19699±997.1	20752±421.1	19502.3±236.7	20917±1094.4	0.02
		**Shannon**	4.84±0.31	5.06±0.54	4.7±0.2	4.28±0.07	4.3±0.02	4.13±0.12	4.3±0.07	4.28±0.08	4.10±0.02	4.18±0.09	0.003
	**Fungi**	**Chao**	1403.3±90.4	1539.6±74.4	1451.3±45.003	1031.3±176.9	1088±27.8	885±21.7	740.6±191.1	984.3±80.8	754.3±22.8	992±17.5	0.002
		**Shannon**	5.26± 0.28	5.44±0.14	5.5±0.04	5.15± 0.26	5.3±0.09	5.46±0.08	5.34± 0.19	5.61±0.04	5.3±0.02	5.5±0.09	0.033

Here, the Kruskal-Wallis rank sum test shows the significance among groups at the α=0.05 level using Dunn's method. OHR: *O. hainanensis* root, MOR: moss+*O. hainanensis* root, M: moss soil, O: *O. hainanensis* soil, ORS: *O. hainanensis* rhizosphere soil, MRS: moss rhizosphere soil, MO: moss+*O. hainanensis* soil, B: bare limestone, MR: moss root, MORS: moss+*O. hainanensis* rhizosphere soil.

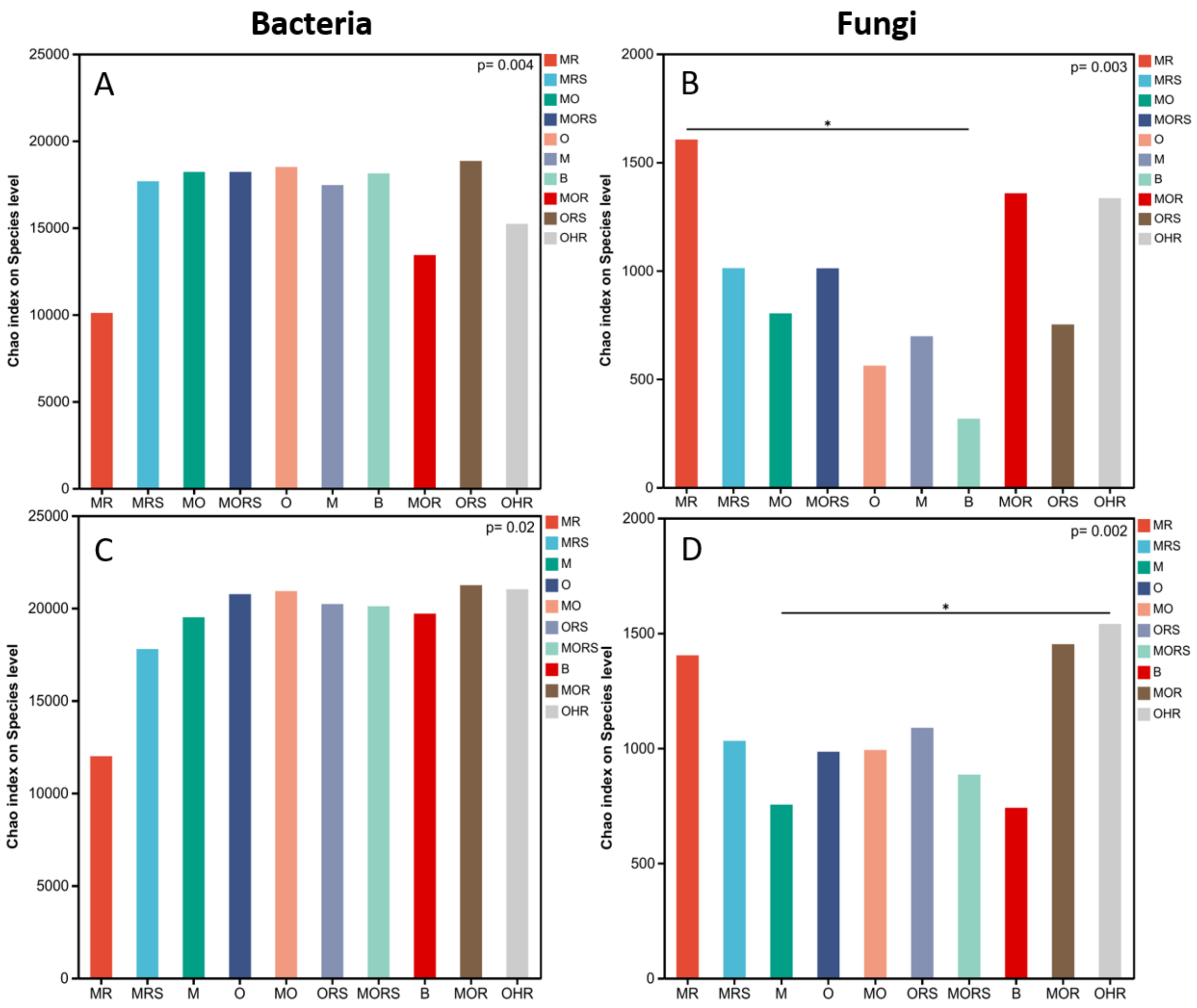

Figure S1. Alpha diversity of the bacterial and fungal community in dry and wet seasons. The Chao index was calculated at the species level based on the phylogenetic distance. Alpha diversity differences among groups were tested using the Kruskal-Wallis rank test (p<0.05). Bacterial and fungal Chao index in the dry season (A, C); bacterial and fungal Chao index in the wet season (B, D).

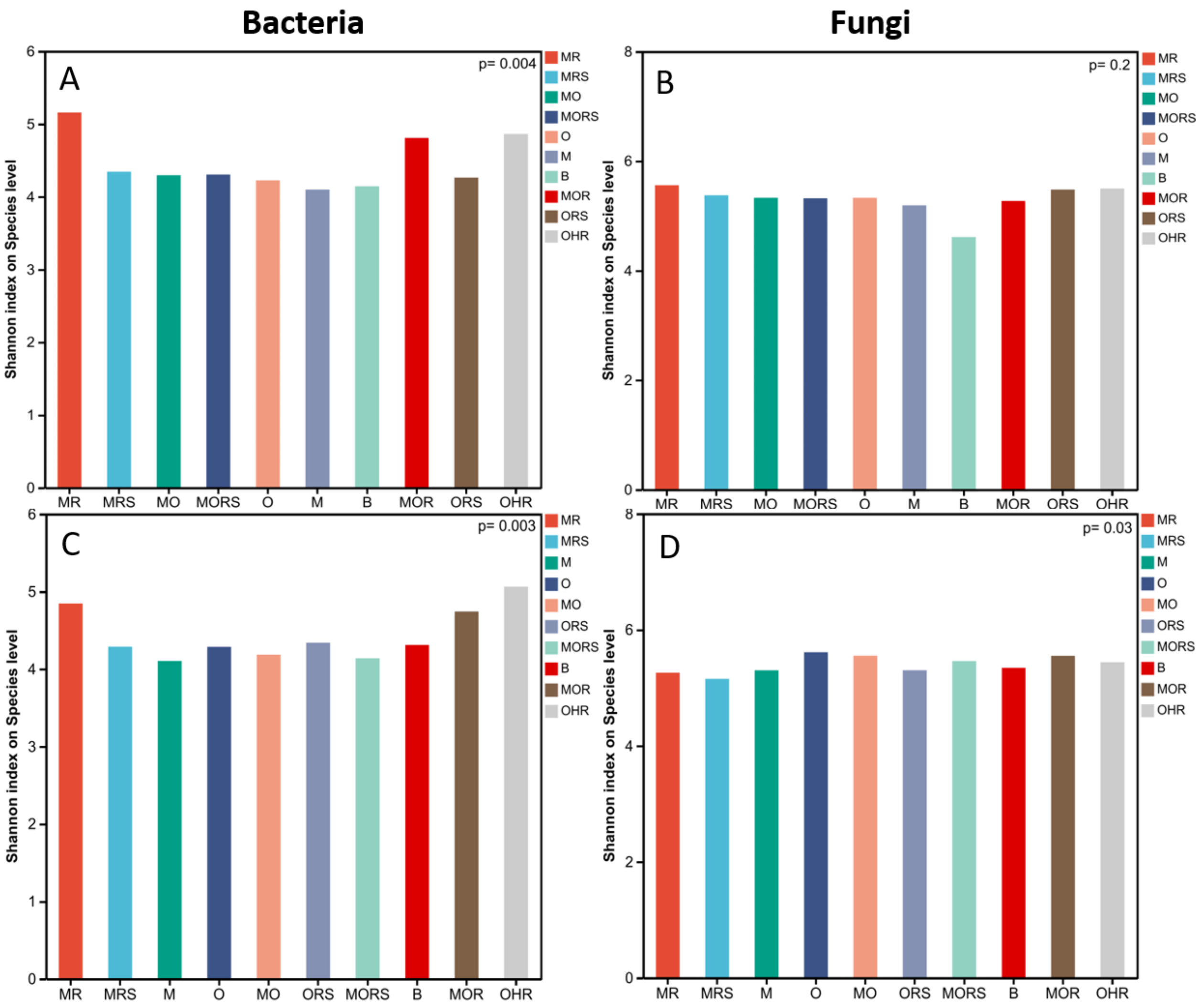

Figure S2. Alpha diversity of the bacterial and fungal community in dry and wet seasons. The Shannon index was calculated at the species level. Differences in Alpha diversity among groups were tested using the Kruskal-Wallis rank sum test (p<0.05). Bacterial and fungal Shannon index in the dry season (A, C); bacterial and fungal Shannon index in the wet season (B, D).

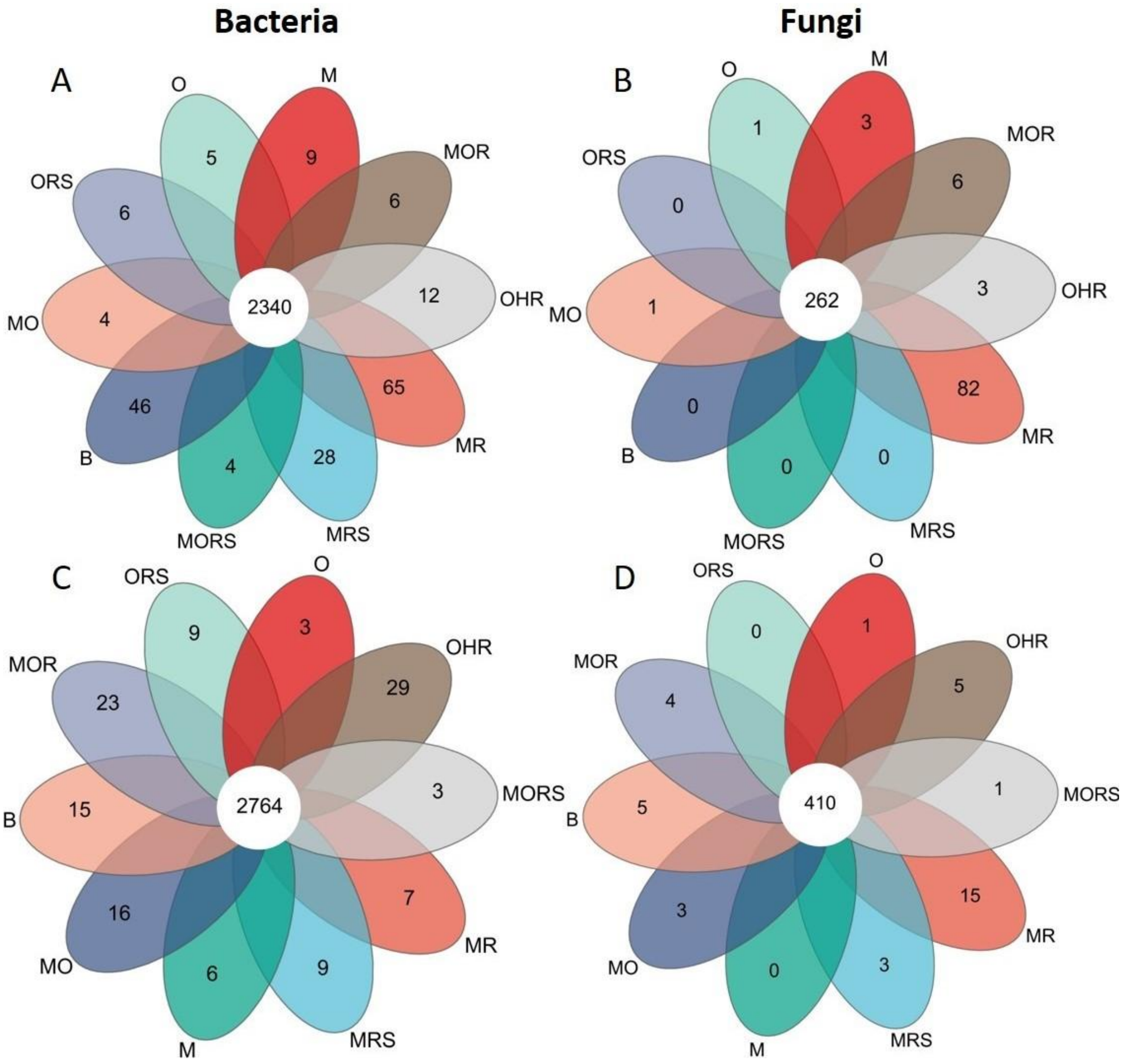

Figure S3. Composition similarities and differences of microorganisms in samples. A, B: Venn diagram of total and shared bacterial and fungal genera in the dry season (A, B); bacterial and fungal total and shared genera in the wet season (C, D). OHR: *O. hainanensis* root, MRS: moss rhizosphere soil, MOR: moss+*O. hainanensis* root, M: moss soil, MR: moss root, O: *O. hainanensis* soil, ORS: *O. hainanensis* rhizosphere soil, MRS: moss rhizosphere soil, MR: moss root, MO: moss+*O. hainanensis* soil, B: bare limestone, MORS: moss+*O. hainanensis* rhizosphere soil.

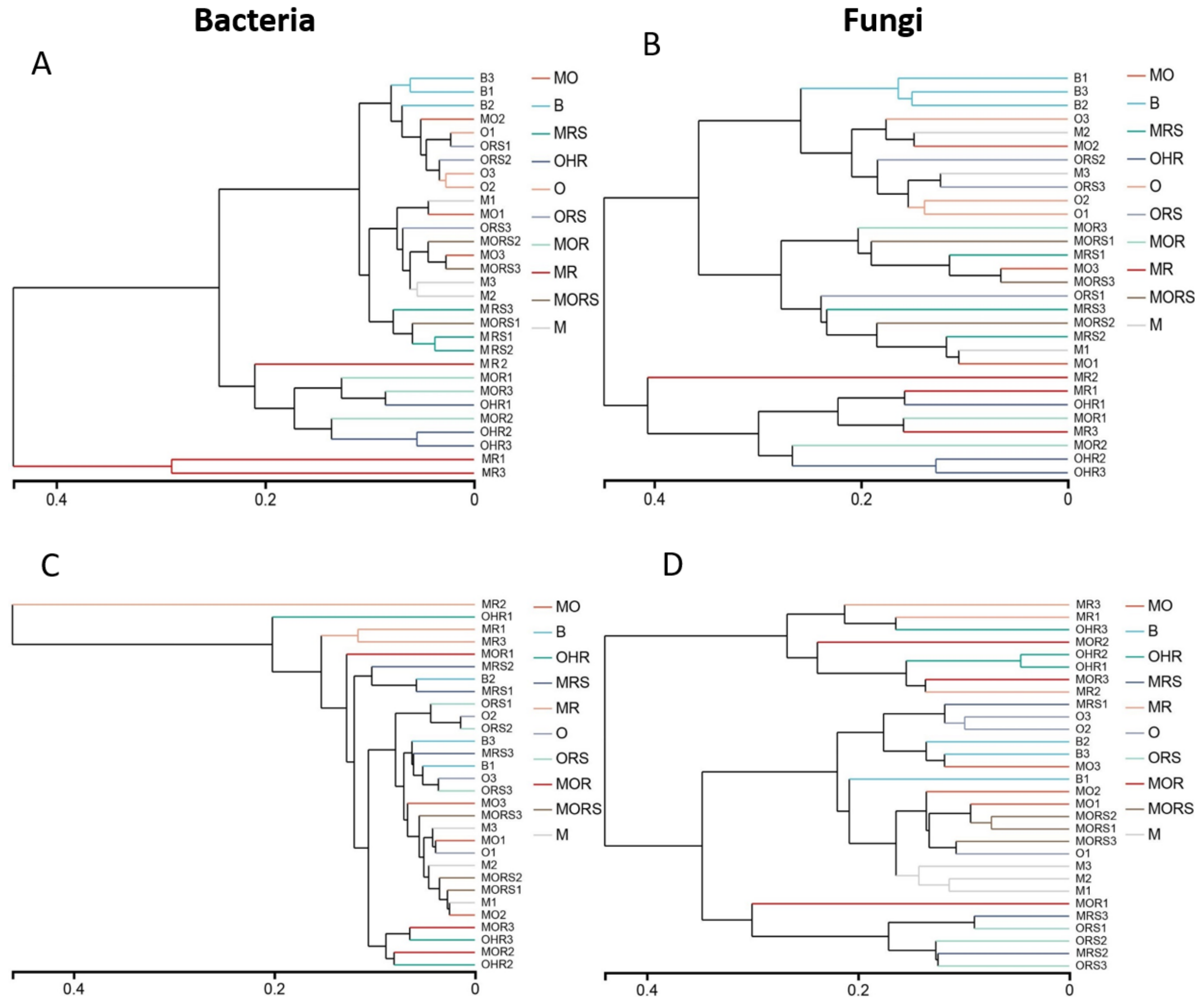

Figure S4. Hierarchical cluster analysis of bacteria and fungi using Bray-Curtis distance. Bacterial and fungal clustering in the dry season (A, C); bacterial and fungal clustering in the wet season (B, D). OHR: *O. hainanensis* root, MRS: moss rhizosphere soil, MOR: moss+*O. hainanensis* root, M: moss soil, MR: moss root, O: *O. hainanensis* soil, ORS: *O. hainanensis* rhizosphere soil, MRS: moss rhizosphere soil, MR: moss root, MO: moss+*O. hainanensis* soil, B: bare limestone, MORS: moss+*O. hainanensis* rhizosphere soil.

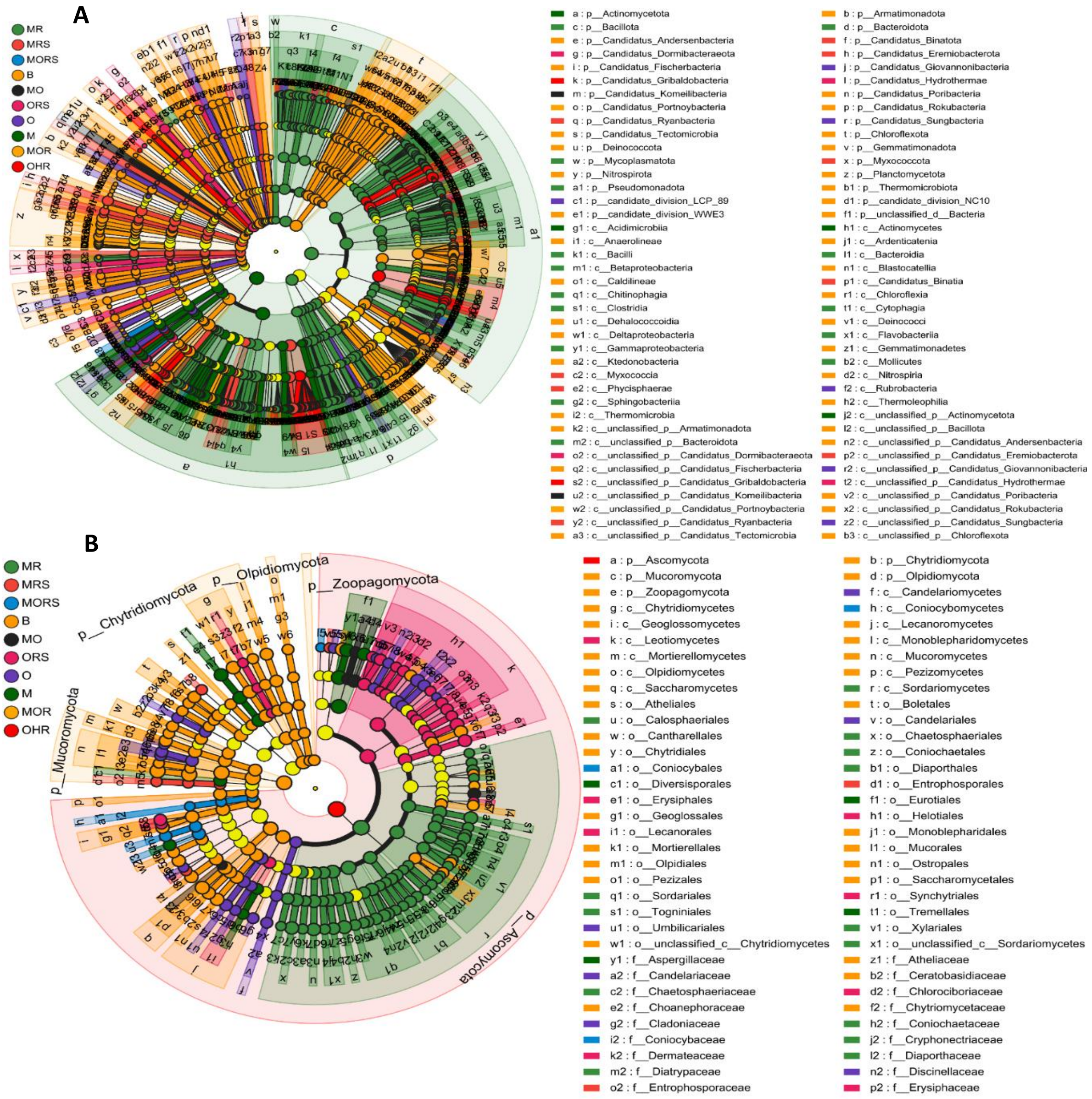

A
MR
MRS
MORS
B
MO
ORS
O
M
MOR
OHR
a : p__Actinomycetota
b : p__Armatimonadota
c : p__Bacillota
d : p__Bacteroidota
e : p__Candidatus_Andersenbacteria
f : p__Candidatus_Binatota
g : p__Candidatus_Dormibacteraeota
h : p__Candidatus_Eremiobacterota
i : p__Candidatus_Fischerbacteria
j : p__Candidatus_Giovannonibacteria
k : p__Candidatus_Gribaldobacteria
l : p__Candidatus_Hydrothermae
m : p__Candidatus_Komeilibacteria
n : p__Candidatus_Poribacteria
o : p__Candidatus_Portnoybacteria
p : p__Candidatus_Rokubacteria
q : p__Candidatus_Ryanbacteria
r : p__Candidatus_Sungbacteria
s : p__Candidatus_Tectomicrobia
t : p__Chloroflexota
u : p__Deinococcota
v : p__Gemmatimonadota
w : p__Mycoplasmatota
x : p__Myxococcota
y : p__Nitrospirota
z : p__Planctomycetota
a1 : p__Pseudomonadota
b1 : p__Thermomicrobiota
c1 : p__candidate_division_LCP_89
d1 : p__candidate_division_NC10
e1 : p__candidate_division_WWE3
f1 : p__unclassified_d__Bacteria
g1 : c__Acidimicrobiia
h1 : c__Actinomycetes
i1 : c__Anaerolineae
j1 : c__Ardenticatenia
k1 : c__Bacilli
l1 : c__Bacteroidia
m1 : c__Betaproteobacteria
n1 : c__Blastocatellia
o1 : c__Caldilineae
p1 : c__Candidatus_Binatia
q1 : c__Chitinophagia
r1 : c__Chloroflexia
s1 : c__Clostridia
t1 : c__Cytophagia
u1 : c__Dehalococcoidia
v1 : c__Deinococci
w1 : c__Deltaproteobacteria
x1 : c__Flavobacteriia
y1 : c__Gammaproteobacteria
z1 : c__Gemmatimonadetes
a2 : c__Ktedonobacteria
b2 : c__Mollicutes
c2 : c__Myxococcia
d2 : c__Nitrospiria
e2 : c__Phycisphaerae
f2 : c__Rubrobacteria
g2 : c__Sphingobacteriia
h2 : c__Thermoleophilia
i2 : c__Thermomicrobia
j2 : c__unclassified_p__Actinomycetota
k2 : c__unclassified_p__Armatimonadota
l2 : c__unclassified_p__Bacillota
m2 : c__unclassified_p__Bacteroidota
n2 : c__unclassified_p__Candidatus_Andersenbacteria
o2 : c__unclassified_p__Candidatus_Dormibacteraeota
p2 : c__unclassified_p__Candidatus_Eremiobacterota
q2 : c__unclassified_p__Candidatus_Fischerbacteria
r2 : c__unclassified_p__Candidatus_Giovannonibacteria
s2 : c__unclassified_p__Candidatus_Gribaldobacteria
t2 : c__unclassified_p__Candidatus_Hydrothermae
u2 : c__unclassified_p__Candidatus_Komeilibacteria
v2 : c__unclassified_p__Candidatus_Poribacteria
w2 : c__unclassified_p__Candidatus_Portnoybacteria
x2 : c__unclassified_p__Candidatus_Rokubacteria
y2 : c__unclassified_p__Candidatus_Ryanbacteria
z2 : c__unclassified_p__Candidatus_Sungbacteria
a3 : c__unclassified_p__Candidatus_Tectomicrobia
b3 : c__unclassified_p__Chloroflexota
B
MR
MRS
MORS
B
MO
ORS
O
M
MOR
OHR
p__Chytridiomycota
p__Olpidiomycota
p__Zoopagomycota
p__Mucoromycota
p__Ascomycota
a : p__Ascomycota
b : p__Chytridiomycota
c : p__Mucoromycota
d : p__Olpidiomycota
e : p__Zoopagomycota
f : c__Candelariomycetes
g : c__Chytridiomycetes
h : c__Coniocybomycetes
i : c__Geoglossomycetes
j : c__Lecanoromycetes
k : c__Leotiomycetes
l : c__Monoblepharidomycetes
m : c__Mortierellomycetes
n : c__Mucoromycetes
o : c__Olpidiomycetes
p : c__Pezizomycetes
q : c__Saccharomycetes
r : c__Sordariomycetes
s : o__Atheliales
t : o__Boletales
u : o__Calosphaeriales
v : o__Candelariales
w : o__Cantharellales
x : o__Chaetosphaeriales
y : o__Chytridiales
z : o__Coniochaetales
a1 : o__Coniocybales
b1 : o__Diaporthales
c1 : o__Diversisporales
d1 : o__Entrophosporales
e1 : o__Erysiphales
f1 : o__Eurotiales
g1 : o__Geoglossales
h1 : o__Helotiales
i1 : o__Lecanorales
j1 : o__Monoblepharidales
k1 : o__Mortierellales
l1 : o__Mucorales
m1 : o__Olpidiales
n1 : o__Ostropales
o1 : o__Pezizales
p1 : o__Saccharomycetales
q1 : o__Sordariales
r1 : o__Synchytriales
s1 : o__Togniniales
t1 : o__Tremellales
u1 : o__Umbilicariales
v1 : o__Xylariales
w1 : o__unclassified_c__Chytridiomycetes
x1 : o__unclassified_c__Sordariomycetes
y1 : f__Aspergillaceae
z1 : f__Atheliaceae
a2 : f__Candelariaceae
b2 : f__Ceratobasidiaceae
c2 : f__Chaetosphaeriaceae
d2 : f__Chlorociboriaceae
e2 : f__Choanephoraceae
f2 : f__Chytriomycetaceae
g2 : f__Cladoniaceae
h2 : f__Coniochaetaceae
i2 : f__Coniocybaceae
j2 : f__Cryphonectriaceae
k2 : f__Dermateaceae
l2 : f__Diaporthaceae
m2 : f__Diatrypaceae
n2 : f__Discinellaceae
o2 : f__Entrophosporaceae
p2 : f__Erysiphaceae

C

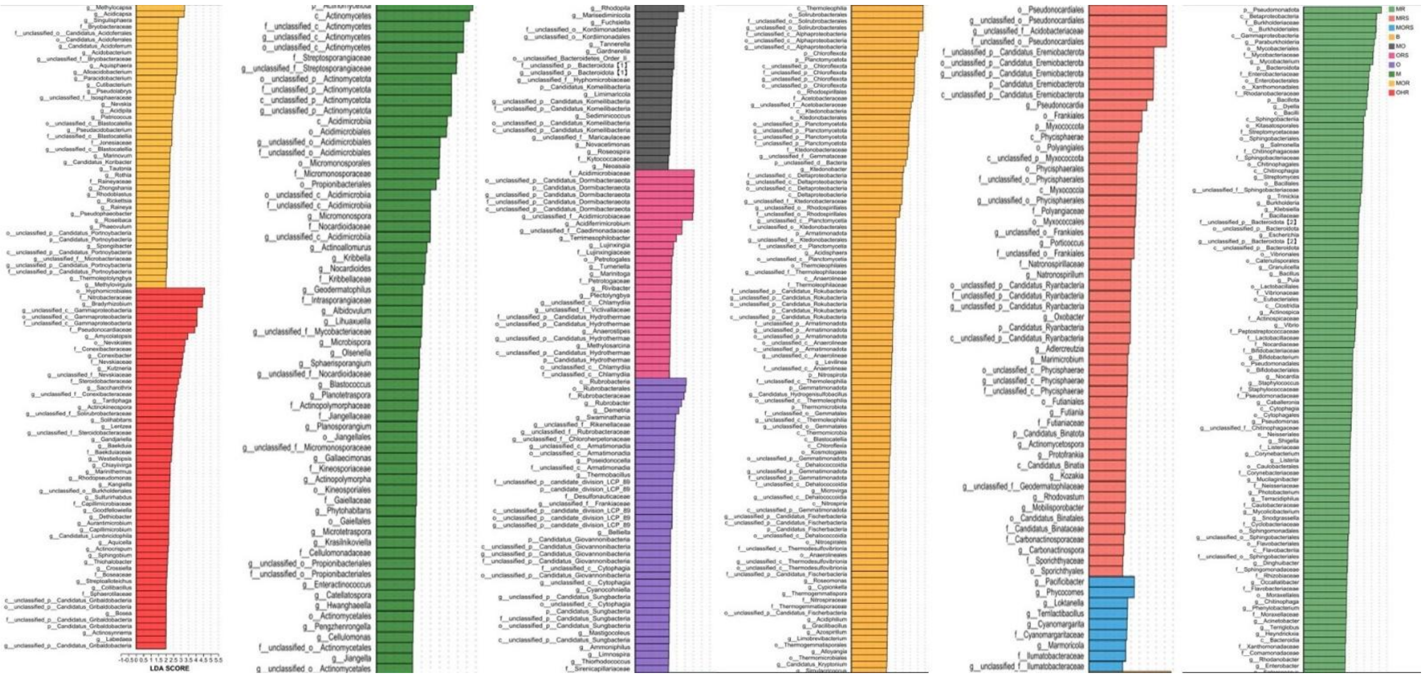

D

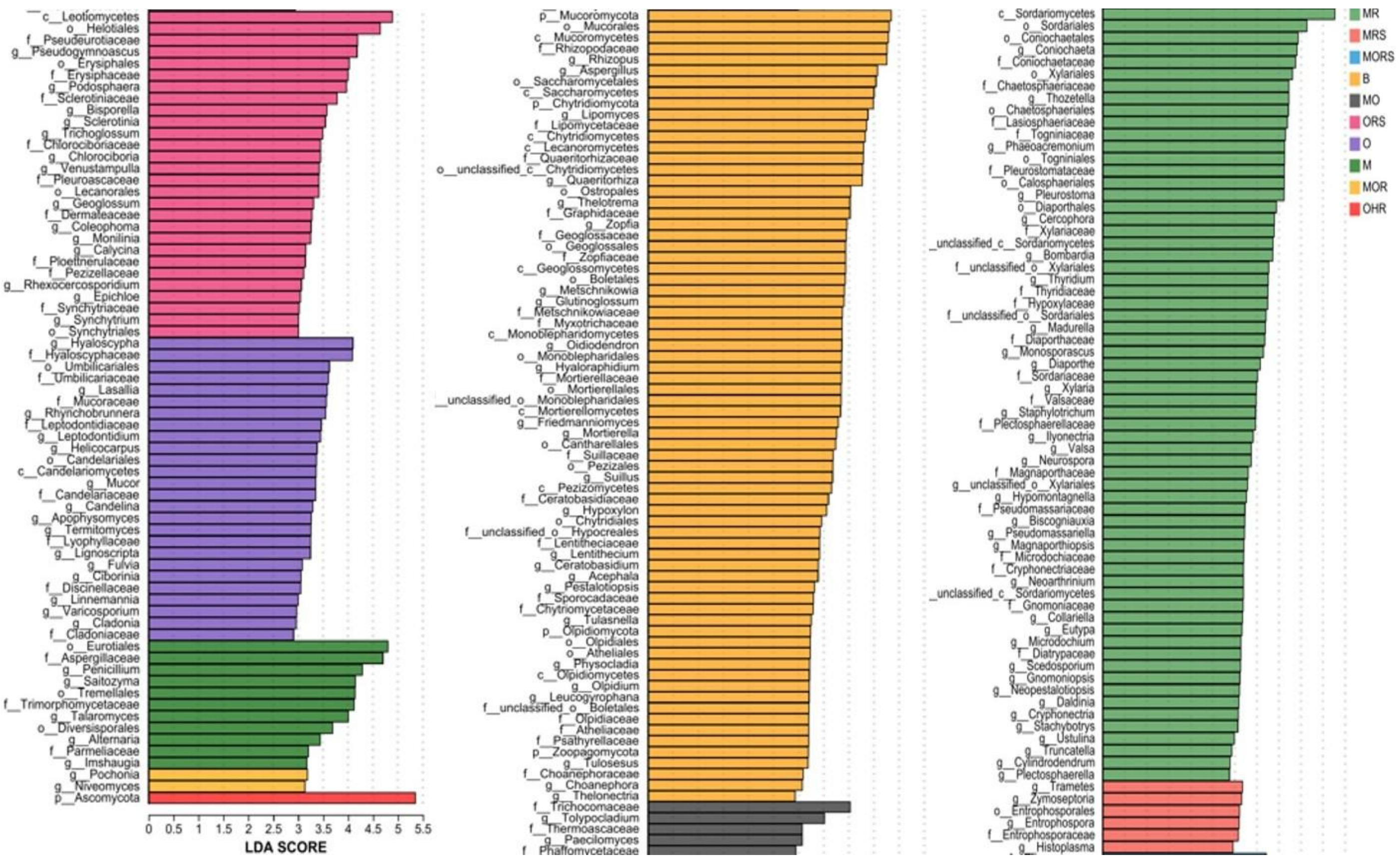

Figure S5A, B, C, D. The LEfSe results revealed bacterial and fungal biomarkers from phylum level to order level that were sensitive to MORS, B, MO, ORS, MR, O, M, MOR, MRS, and OHR in the dry season. There are five circular rings in the cladogram, and each circular ring deposits all taxa within a taxonomic level; the circular ring from inside to outside represents phylum, class, and order, or with an additional family, respectively. The node on the circular ring represents a taxon affiliating within the taxonomic level. Taxa with significantly higher relative abundance in a certain group were color-coded within the cladogram according to the bacterial and fungal Ribosomal reference (PR2) taxonomy.

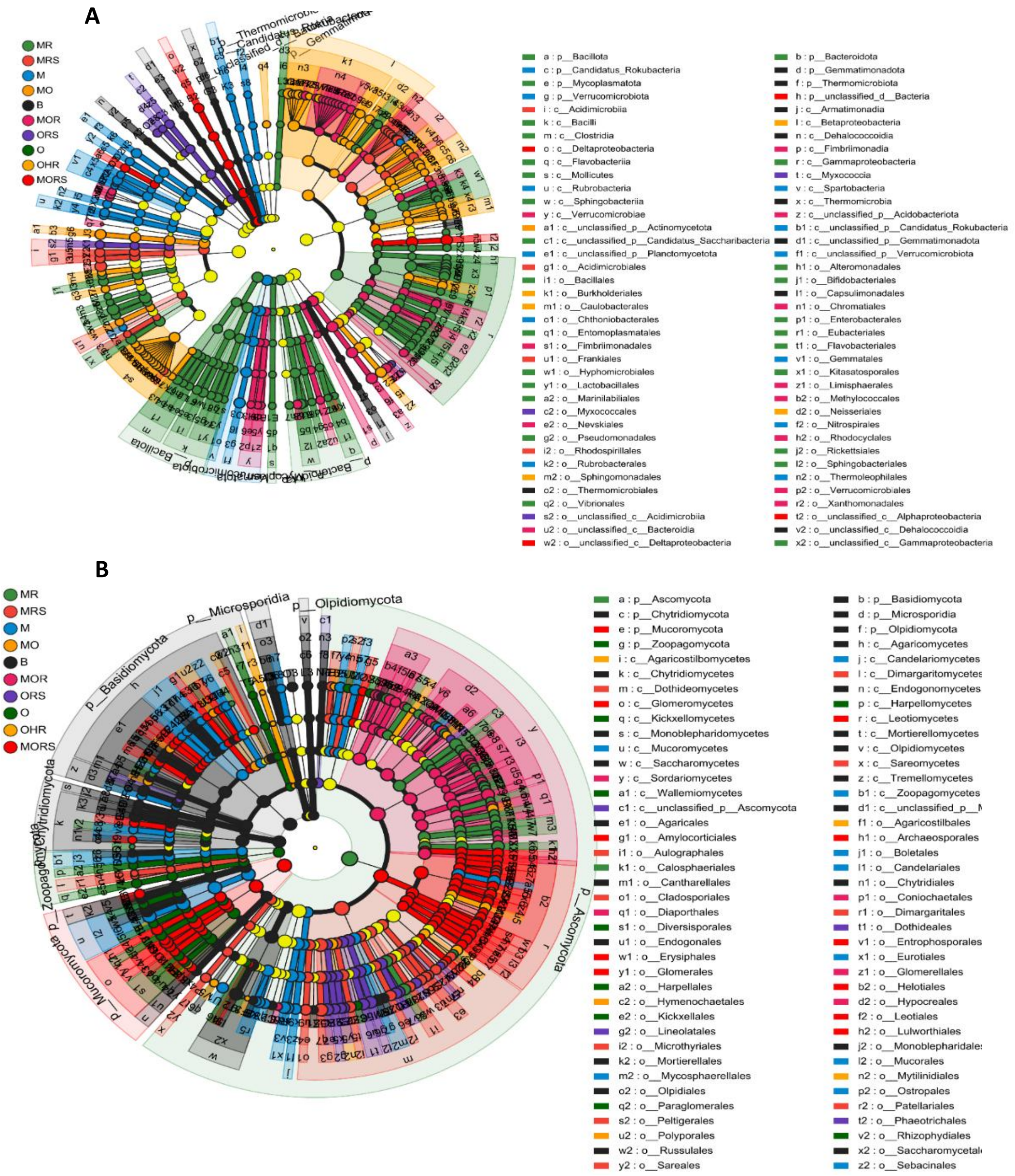
A
MR
MRS
M
MO
B
MOR
ORS
O
OHR
MORS
a : p__Bacillota
c : p__Candidatus_Rokubacteria
e : p__Mycoplasmatota
g : p__Verrucomicrobiota
i : c__Acidimicrobiia
k : c__Bacilli
m : c__Clostridia
o : c__Deltaproteobacteria
q : c__Flavobacteriia
s : c__Mollicutes
u : c__Rubrobacteria
w : c__Sphingobacteriia
y : c__Verrucomicrobiae
a1 : c__unclassified_p__Actinomycetota
c1 : c__unclassified_p__Candidatus_Saccharibacteria
e1 : c__unclassified_p__Planctomycetota
g1 : o__Acidimicrobiales
i1 : o__Bacillales
k1 : o__Burkholderiales
m1 : o__Caulobacterales
o1 : o__Chthoniobacterales
q1 : o__Entomoplasmatales
s1 : o__Fimbriimonadales
u1 : o__Frankiales
w1 : o__Hyphomicrobiales
y1 : o__Lactobacillales
a2 : o__Marinilabiliales
c2 : o__Myxococcales
e2 : o__Nevskiales
g2 : o__Pseudomonadales
i2 : o__Rhodospirillales
k2 : o__Rubrobacterales
m2 : o__Sphingomonadales
o2 : o__Thermomicrobiales
q2 : o__Vibrionales
s2 : o__unclassified_c__Acidimicrobiia
u2 : o__unclassified_c__Bacteroidia
w2 : o__unclassified_c__Deltaproteobacteria
b : p__Bacteroidota
d : p__Gemmatimonadota
f : p__Thermomicrobiota
h : p__unclassified_d__Bacteria
j : c__Armatimonadia
l : c__Betaproteobacteria
n : c__Dehalococcoidia
p : c__Fimbriimonadia
r : c__Gammaproteobacteria
t : c__Myxococcia
v : c__Spartobacteria
x : c__Thermomicrobia
z : c__unclassified_p__Acidobacteriota
b1 : c__unclassified_p__Candidatus_Rokubacteria
d1 : c__unclassified_p__Gemmatimonadota
f1 : c__unclassified_p__Verrucomicrobiota
h1 : o__Alteromonadales
j1 : o__Bifidobacteriales
l1 : o__Capsulimonadales
n1 : o__Chromatiales
p1 : o__Enterobacterales
r1 : o__Eubacteriales
t1 : o__Flavobacteriales
v1 : o__Gemmatales
x1 : o__Kitasatosporales
z1 : o__Limisphaerales
b2 : o__Methylococcales
d2 : o__Neisseriales
f2 : o__Nitrospirales
h2 : o__Rhodocyclales
j2 : o__Rickettsiales
l2 : o__Sphingobacteriales
n2 : o__Thermoleophilales
p2 : o__Verrucomicrobiales
r2 : o__Xanthomonadales
t2 : o__unclassified_c__Alphaproteobacteria
v2 : o__unclassified_c__Dehalococcoidia
x2 : o__unclassified_c__Gammaproteobacteria
B
MR
MRS
M
MO
B
MOR
ORS
O
OHR
MORS
p__Basidiomycota
p__Microsporidia
p__Olpidiomycota
p__Ascomycota
p__Mucoromycota
a : p__Ascomycota
c : p__Chytridiomycota
e : p__Mucoromycota
g : p__Zoopagomycota
i : c__Agaricostilbomycetes
k : c__Chytridiomycetes
m : c__Dothideomycetes
o : c__Glomeromycetes
q : c__Kickxellomycetes
s : c__Monoblepharidomycetes
u : c__Mucoromycetes
w : c__Saccharomycetes
y : c__Sordariomycetes
a1 : c__Wallemiomycetes
c1 : c__unclassified_p__Ascomycota
e1 : o__Agaricales
g1 : o__Amylocorticiales
i1 : o__Aulographales
k1 : o__Calosphaeriales
m1 : o__Cantharellales
o1 : o__Cladosporiales
q1 : o__Diaporthales
s1 : o__Diversisporales
u1 : o__Endogonales
w1 : o__Erysiphales
y1 : o__Glomerales
a2 : o__Harpellales
c2 : o__Hymenochaetales
e2 : o__Kickxellales
g2 : o__Lineolatales
i2 : o__Microthyriales
k2 : o__Mortierellales
m2 : o__Mycosphaerellales
o2 : o__Olpidiales
q2 : o__Paraglomerales
s2 : o__Peltigerales
u2 : o__Polyporales
w2 : o__Russulales
y2 : o__Sareales
b : p__Basidiomycota
d : p__Microsporidia
f : p__Olpidiomycota
h : c__Agaricomycetes
j : c__Candelariomycetes
l : c__Dimargaritomycetes
n : c__Endogonomycetes
p : c__Harpellomycetes
r : c__Leotiomycetes
t : c__Mortierellomycetes
v : c__Olpidiomycetes
x : c__Sareomycetes
z : c__Tremellomycetes
b1 : c__Zoopagomycetes
f1 : o__Agaricostilbales
h1 : o__Archaeosporales
j1 : o__Boletales
l1 : o__Candelariales
n1 : o__Chytridiales
p1 : o__Coniochaetales
r1 : o__Dimargaritales
t1 : o__Dothideales
v1 : o__Entrophosporales
x1 : o__Eurotiales
z1 : o__Glomerellales
b2 : o__Helotiales
d2 : o__Hypocreales
f2 : o__Leotiales
h2 : o__Lulworthiales
l2 : o__Mucorales
n2 : o__Mytilinidiales
p2 : o__Ostropales
r2 : o__Patellariales
t2 : o__Phaeotrichales
v2 : o__Rhizophydiales
z2 : o__Sebacinales

C

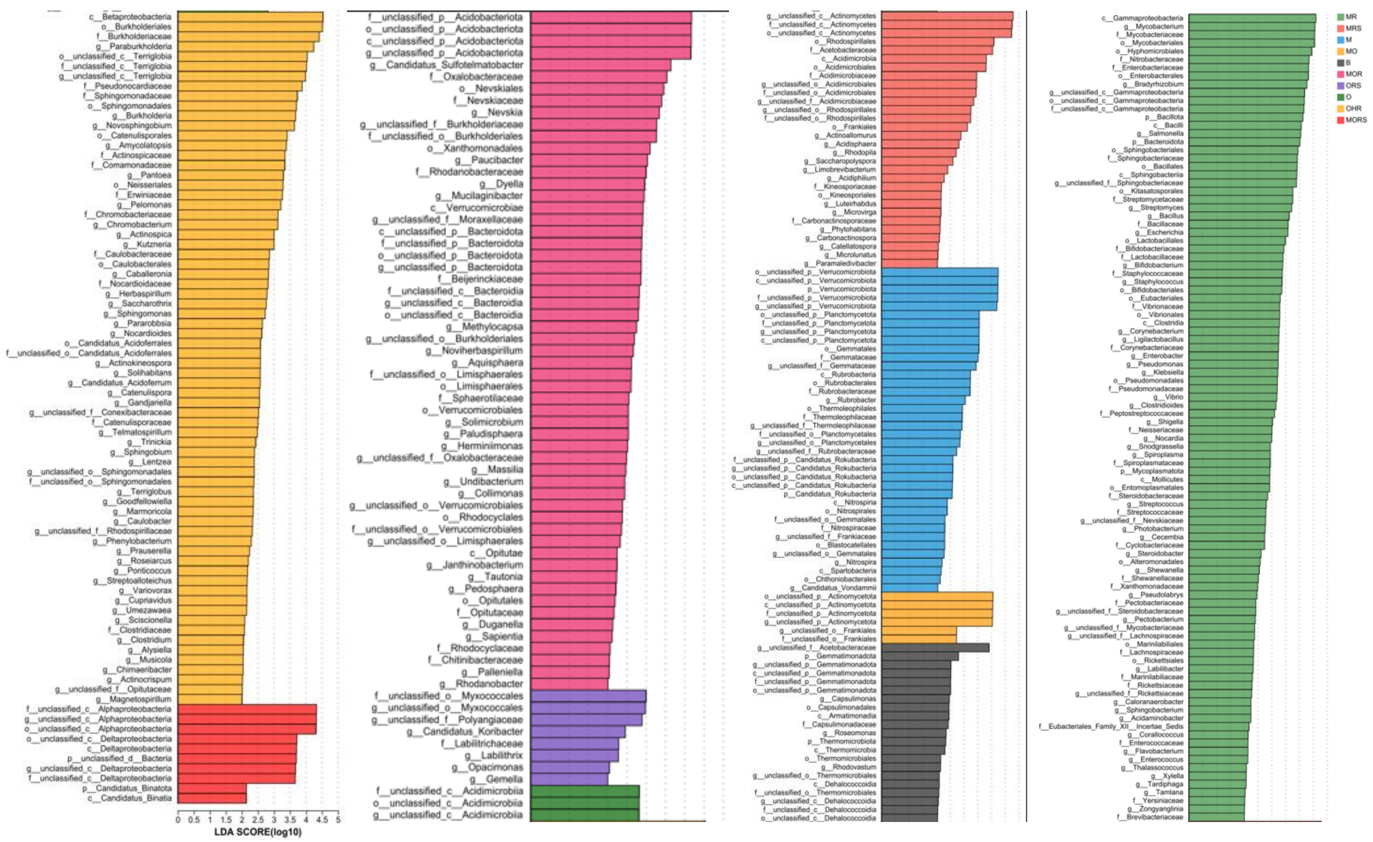
0
0.5
1
1.5
2
2.5
3
3.5
4
4.5
5
LDA SCORE(log10)
MR
MRS
M
MO
B
MOR
ORS
O
OHR
MORS

D

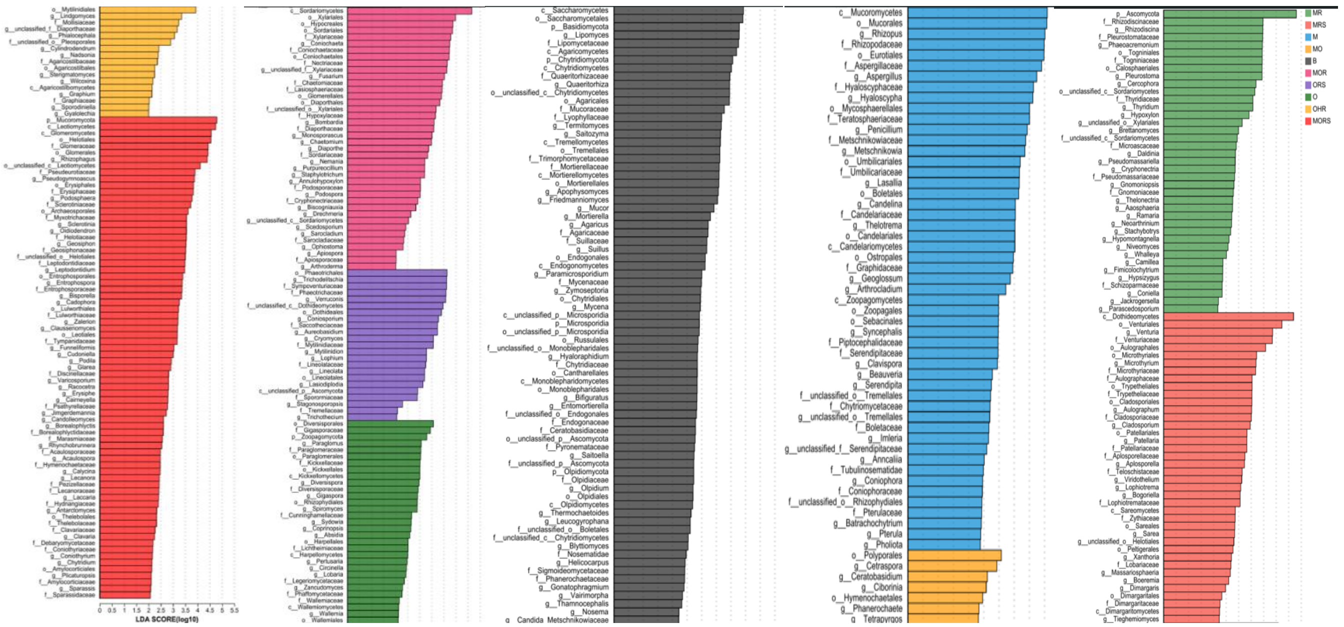

Figure S6A, B, C, D. The LEfSe results revealed bacterial and fungal biomarkers from phylum level to order level that were sensitive to MORS, B, MO, ORS, MR, O, M, MOR, MRS, and OHR in the wet season. There are five circular rings in the cladogram, and each circular ring deposits all taxa within a taxonomic level; the circular ring from inside to outside represents phylum, class, and order, or with an additional family, respectively. The node on the circular ring represents a taxon affiliating within the taxonomic level. Taxa with significantly higher relative abundance in a certain group were color-coded within the cladogram according to the bacterial and fungal Ribosomal reference (PR2) taxonomy.

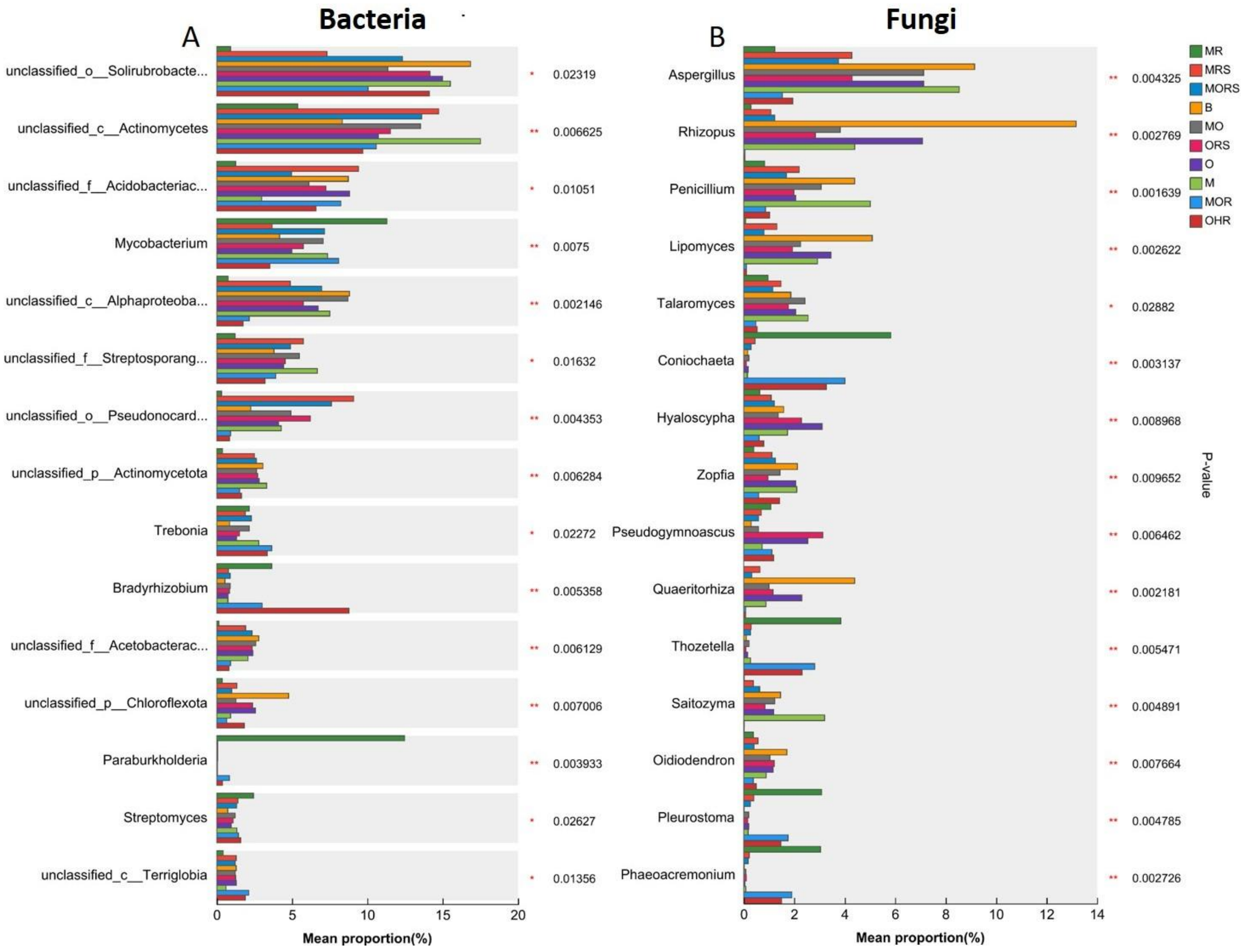

Figure S7A, B. A Multigroup comparison test was conducted using the Kruskal-Wallis rank test of bacterial (A) and Fungal (B) groups in the dry season. The bar chart showed the difference in the average relative abundance of the same species between different groups and labeled whether the difference was significant (*P*-value, asterisk indicates significant difference). The significance of the differences between the same species in multiple groups was visually demonstrated. The ordinate represents the name of the species at different taxonomic levels, the abscissa represents the percentage value of the abundance of a species in the sample, and different colors represent different groups. $0.01<p<=0.05$ *, $0.001<p<=0.01$ **, $p<=0.001$ ***.

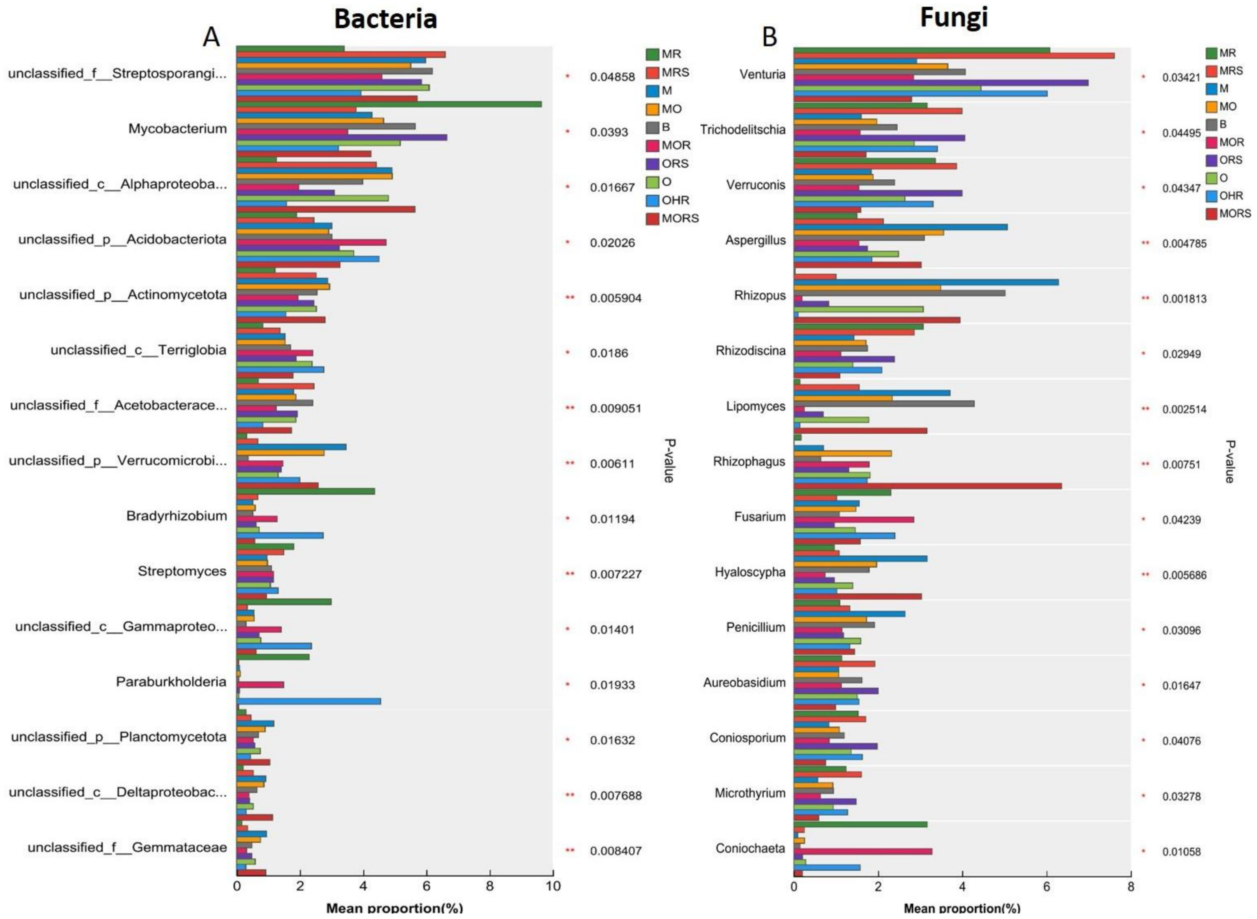

Figure S8A, B. A Multigroup comparison test was conducted using the Kruskal-Wallis rank test of bacterial (A) and Fungal (B) groups in the wet season. The bar chart showed the difference in the average relative abundance of the same species between different groups and labeled whether the difference was significant (P-value, asterisk indicates significant difference). The significance of the differences between the same species in multiple groups was visually demonstrated. The ordinate represents the name of the species at different taxonomic levels, the abscissa represents the percentage value of the abundance of a species in the sample, and different colors represent different groups. $0.01<p<=0.05$ *, $0.001<p<=0.01$ **, $p<=0.001$ ***.
